# Adhesion Molecules Associated with Female Genital Tract Infection

**DOI:** 10.1371/journal.pone.0156605

**Published:** 2016-06-07

**Authors:** Jamal Qualai, Jon Cantero, Lin-Xi Li, José Manuel Carrascosa, Eduard Cabré, Olga Dern, Lauro Sumoy, Gerard Requena, Stephen J. McSorley, Meritxell Genescà

**Affiliations:** 1 Mucosal Immunology Unit, Institut d’Investigació en Ciències de la Salut Germans Trias i Pujol (IGTP), AIDS Research Institute IrsiCaixa-HIVACAT, Can Ruti Campus, Badalona, Spain; 2 Department of Microbiology and Immunology, University of Arkansas for Medical Sciences, Little Rock, Arkansas, United States of America; 3 Department of Dermatology, University Hospital “Germans Trias i Pujol,” Badalona, Universitat Autònoma de Barcelona, Spain; 4 Department of Gastroenterology, University Hospital “Germans Trias i Pujol,” Can Ruti Campus, Badalona, Catalonia, Spain; 5 Centro de Investigación Biomédica en Red de Enfermedades Hepáticas y Digestivas (CIBERehd), Madrid, Spain; 6 Atenció Salut Sexual i Reproductiva (ASSIR), Centre d'Atenció Primària (CAP) Sant Fèlix, Institut Català de la Salut (ICS), Sabadell, Spain; 7 Genomics and Bioinformatics Group, Institute for Predictive and Personalized Medicine of Cancer (IMPPC), Can Ruti Campus, Badalona, Spain; 8 Flow Cytometry Unit, Institut d’Investigació en Ciències de la Salut Germans Trias i Pujol, Badalona, Spain; 9 Center for Comparative Medicine (CCM), Department of Anatomy, Physiology and Cell Biology, School of Veterinary Medicine, University of California Davis, Davis, California, United States of America; University of Pittsburgh Center for Vaccine Research, UNITED STATES

## Abstract

Efforts to develop vaccines that can elicit mucosal immune responses in the female genital tract against sexually transmitted infections have been hampered by an inability to measure immune responses in these tissues. The differential expression of adhesion molecules is known to confer site-dependent homing of circulating effector T cells to mucosal tissues. Specific homing molecules have been defined that can be measured in blood as surrogate markers of local immunity (e.g. α4β7 for gut). Here we analyzed the expression pattern of adhesion molecules by circulating effector T cells following mucosal infection of the female genital tract in mice and during a symptomatic episode of vaginosis in women. While CCR2, CCR5, CXCR6 and CD11c were preferentially expressed in a mouse model of *Chlamydia* infection, only CCR5 and CD11c were clearly expressed by effector T cells during bacterial vaginosis in women. Other homing molecules previously suggested as required for homing to the genital mucosa such as α4β1 and α4β7 were also differentially expressed in these patients. However, CD11c expression, an integrin chain rarely analyzed in the context of T cell immunity, was the most consistently elevated in all activated effector CD8^+^ T cell subsets analyzed. This molecule was also induced after systemic infection in mice, suggesting that CD11c is not exclusive of genital tract infection. Still, its increase in response to genital tract disorders may represent a novel surrogate marker of mucosal immunity in women, and warrants further exploration for diagnostic and therapeutic purposes.

## Introduction

Female genital tract (FGT) infections, including common sexually transmitted infections (STI), seriously compromise the health of women. Worldwide, more than 340 million new cases of treatable STI occur each year and they are estimated to be the leading cause of morbidity in women in developing countries [1]. Furthermore, pre-existing FGT infections affect the development and pathogenesis of other STI, as occurs with the pro-inflammatory environment generated by bacterial vaginosis (BV) and the enhancement of human immunodeficiency virus (HIV) replication [2]. The long-term consequences of STI, including pelvic inflammatory disease, cancer, infertility, stillbirth, etc. not only are highly relevant at the social and health level, but also have a major economic impact.

Although effective vaccines exist for human papilloma virus and hepatitis B virus, efforts to develop vaccines against herpes simplex virus type 2 (HSV-2), HIV and bacterial STI have been hampered by an inability to effectively measure immune responses in the genital tract. Such vaccines need to be able to generate robust immune responses at site of potential exposure in order to provide rapid control of primary infection [3, 4]. Mucosal T cells and, notably, cytotoxic T lymphocytes play a critical role in the clearance of sexually transmitted pathogens [4]. For instance, studies in human have confirmed the association of T cell-mediated immunity with clearance of *Chlamydia* infection [5] and susceptibility to re-infection [6]. Moreover, the presence of antiviral effector CD8^+^ T cells in the vagina of immunized monkeys correlates with protection from uncontrolled viremia after pathogenic challenge with simian immunodeficiency virus [7]. In these models of genital infection, the induction of effector memory T (T_EM_) cells and antibodies that are able to mount fast responses upon re-challenge is critical to control the pathogen. However, current assays used to understand the magnitude and quality of immune responses in the FGT rely primarily on blood samples and thus provide an incomplete picture of localized immune control.

The capacity of distinct subsets of antigen-experienced lymphocytes to traffic preferentially into specific compartments is termed homing. T_EM_ cell entry into inflamed non-lymphoid tissues is an active process involving members of the integrin, selectin-ligand and chemokine-receptor families, which mediate selective interactions of circulating lymphocytes with the specialized vascular endothelium [8]. While some adhesion molecules are enriched for a given tissue, e.g. α4β7 integrin and CC chemokine receptor CCR9 are associated with homing of T cells to the gut and cutaneous lymphocyte-associated antigen (CLA) and CCR4/CCR10 with T cell homing to the skin, other molecules are specialized for tissue-inflammatory functions to multiple tissues, such as CXCR3 or αLβ2 [9, 10]. Importantly, many properties that enable T cells to traffic to specific locations are programmed during the early stages of the infection [11]. Analysis of blood samples during the primary immune response to yellow fever immunization in humans suggests that human virus-specific CD8^+^ T cells express a dynamic pattern of homing molecules early after immune activation [12]. Thus, analysis of lymphocytes in blood will not reflect the quantity/quality of non-recirculating resident memory T cells [11], and sampling directly from mucosal surfaces will be required to define correlates of protection after vaccination. However, after activation, there is a window of opportunity to examine circulating lymphocytes in blood as they are homing to a specific mucosal tissue.

In contrast to the gastrointestinal tract or the skin, our understanding of homing receptors that are required for cells migrating to the FGT is limited. α4β7 was proposed to recruit CD4^+^ T cells to the vaginal mucosa of mice infected with *Chlamydia* [13]. Yet, a recent paper demonstrated that this integrin is not necessary for CD4^+^ T cell-mediated protection against *Chlamydia trachomatis* infection, while α4β1 appears to drive homing of protective cells into the murine upper genital tract [14]. Other papers using animal models support these findings [15] and expression of vascular cell adhesion molecule-1, which binds this integrin, has been detected in the human vagina [16, 17]. Nonetheless, another recent paper demonstrated that circulating CD4^+^ T cells from asymptomatic HSV-2-infected patients express higher levels of α4β7 than uninfected patients [18]. Lastly, other investigators have described homing receptors that are shared between immune cells migrating to the skin and the FGT [16, 19]. Here, we hypothesized that defining the homing profiles of lymphocytes migrating towards the genital tract could potentially be used as a surrogate marker of FGT immunity. We found that T_EM_ cells from mice intravaginally-infected with *Chlamydia muridarum* expressed high levels of CCR2, CCR5, CXCR6 and CD11c. When comparing these homing profiles to women with different mucosal or skin disorders, unique features were detected in each of the cohorts, compared to healthy patients. Of particular interest, CD11c was strikingly increased in CD8^+^ T_EM_ cells from patients with BV and thus could serve as a novel indirect marker of FGT immunity.

## Materials and Methods

### Ethics statement

All mice were maintained in accordance with the recommendations of the Association for Assessment and Accreditation of Laboratory Animal Care International Standards and with the recommendations in the Guide for the Care and Use of Laboratory Animals of the National Institutes of Health. The Institutional Animal Use and Care Committee of the University of California, Davis, approved these experiments (Protocol # 18299).

Informed written consent was obtained from all participants and the study protocol and questionnaire was approved by the University Hospital Germans Trias i Pujol (HUGTP, Badalona, Spain) Clinical Research Ethics Committee (reference # EO-11-074). The study was undertaken in accordance with the Declaration of Helsinki and the requirements of Good Clinical Practice.

### Animal model

*Chlamydia muridarum* strain Weiss was purchased from ATCC (Manassas, VA) and propagated in HeLa 229 cells in Dulbecco's modified Eagle's medium (Life Technologies, Grand Island, NY) supplemented with 10% fetal bovine serum (FBS). *C*. *muridarum* elementary bodies (EBs) were purified by discontinuous density gradient centrifugation as previously described and stored at -80°C [20]. The number of inclusion-forming units of purified EBs was determined by infection of HeLa 229 cells and enumeration of inclusions that were stained with anti-*Chlamydia* major outer membrane protein antibody (a kind gift from Dr. Harlan Caldwell). Eight weeks old C57BL/6 mice were purchased from The Jackson Laboratory (Bar Harbor, ME). For systemic infection, mice were intravenously (IV) injected in the lateral tail vein with 1x10^5^ C. muridarum. For vaginal infection, estrus was synchronized by subcutaneous injection of 2.5 mg medroxyprogesterone acetate (Greenstone, NJ). Seven days after, 1x10^5^ C. muridarum in 5μL sucrose/phosphate/glutamate buffer were deposited directly into the vaginal vaults with a blunted pipet tip [21]. Seven, 10 and 14 days post-infection, blood was collected by retro-orbital bleeding. Immediately after collection, blood samples for cell sorting and validation analyses were air-shipped from the laboratory of Dr. McSorley (CCM, UC Davis, CA) to the laboratory of Dr. Genescà (IGTP, Badalona, Spain). Samples arrived refrigerated and in good conditions 48 hours later and were immediately processed.

### Cell sorting

Circulating T_EM_ cells were sorted from six 7 days *Chlamydia*-infected mice (VAG group) and six contemporary sham-treated mice (control group). Blood samples (~500μl) were immediately lysed using an in-house red blood cell lysis buffer. After washing, cells were stained with antibodies against CD3-Vioblue (145-2C11), CD62L-PE (MEL-14-H2.100) and CD44-FITC (IM7.8.1; all from Miltenyi Biotec, Madrid, Spain). Cells suspended in cold FACS flow buffer (0.5% FBS-PBS with 0.5mM EDTA) were immediately sorted into 350μl of chilled RLT buffer (QIAGEN, Valencia, CA) using a BD FACSAria^™^ Cell Sorter (Flow Cytometry Platform, IGTP). Purity of sorted CD62L^-^ CD44^+^ activated T_EM_ cells was >99%. Once sorted, samples were mixed for a minute and after a short spin, the volume of RLT was adjusted (3.5:1 ratio of RLT to sheath fluid) using filtered tips. Samples were mixed and spun again and immediately frozen at -80°C.

### Gene expression analyses

Total RNA was isolated from cells using RNA easy Mini kit (QIAGEN). After qualitative assessment of RNA integrity, samples were amplified using Whole Transcriptome Amplification 2 (Sigma-Aldrich, Madrid, Spain). We chose four samples in each group that qualified for microarray analyses, yet one sample from the VAG and control group were discarded after as outliers, thus microarray analyses were performed in n = 3 for the VAG group and n = 3 for the control group (which were actually contemporary samples). The number of sorted cells in each group was similar (15,207 ± 2,339 cells for the VAG group and 20,793 ± 2,443 cells for the control group; not significant).

Affymetrix microarray hybridization was performed using the Mouse Genome 430 PM Strip platform. Images intensities were extracted using GeneAtlas System software (Affymetrix), normalized and summarized using Robust Multi-array Average algorithm. Differential gene expression analysis was assessed by fitting to an empirical Bayesian linear model. Statistical significance in differential gene expression was computed with a false discovery rate multiple testing adjustment correction. For this analysis we used the Limma package and the R statistical programming environment [22]. The ‘compute overlaps’ function in the ‘investigate gene sets web application (http://www.broadinstitute.org/gsea/msigdb/annotate.jsp) of the Molecular Signatures Database v4.0 (MaSigDB) was used to explore functions enriched among the top regulated genes defined as having a nominal (non-adjusted) p-value <0.005. The lists of gene symbols up-regulated and down-regulated in infected versus uninfected samples were scrutinized for significant overlaps with pathway (C2: CP, KEGG) and immunologic signatures (C7) in MaSigDB [23].

### Flow cytometry validation analyses in mice

Blood samples (~500μl) were immediately lysed, washed, suspended in PBS and incubated with Aqua vital dye to distinguish live from dead cells (Invitrogen, Burlington, ON, Canada). Following two more washes, cells were suspended in washing and staining buffer (1% bovine serum albumin-PBS) and incubated for 20 minutes with the following cocktail of pre-titrated anti-mouse antibodies: CD3-Vioblue (145-2C11), CD4-APC-H7 (GK1.5), CD62L-PE (MEL-14-H2.100) (Miltenyi Biotec), CD44-Brilliant Violet 570 (IM7; BioLegend, San Diego, CA), CD11c-PE-Cy7 (HL3; BD Biosciences) and CCR5-FITC (CTC5), CXCR6-PerCP (221002) and CCR2-APC (475301) (R&D Systems Inc., Minneapolis, MN). All events were acquired in a BD FACSAria^™^ Cell Sorter and analyzed with FlowJo vX.0.7 software (TreeStar, Ashland, OR).

### Participant enrolment and inclusion criteria for human samples

All participants for this study (20–40 year old women) were classified in the following different cohorts: normal donors (ND, n = 13, median age of 25 years with interquartile range (IQR) of 23–28 years), psoriasis (PS, n = 5, median of 35 with IQR 30–37), ulcerative colitis (UC, n = 4, median of 26 with IQR 22–38), and BV (n = 5, median of 30 with IQR 25–36). Healthy ND volunteers were recruited from the clinical trials unit of the University Hospital Germans Trias i Pujol (HUGTP, Badalona, Spain). Patient recruitment for the PS and UC cohorts was carried in the corresponding services of Dermatology and Gastroenterology of the HUGTP. BV patients were recruited at the Unit of Attention to Sexual and Reproductive Health from the Primary Health Care centers of Sabadell and Cerdanyola in Catalonia (Spain).

Participants completed a questionnaire in order to detect possible exclusion criteria (other chronic or acute diseases, allergies or infections, immunosuppressive treatment, etc.) and register menstrual cycle and birth control data. Patients included in each cohort presented either an active burst (for PS and UC groups) or symptoms compatible with a recent infection within the last 7 days (for BV group). Inclusion criteria for the PS group consisted on women with acute psoriasis vulgaris that suffered a relapse of less than two weeks, with no treatment for at least six weeks. UC subjects were women with active ulcerative colitis: the extent of pathology was defined based on the Montreal classification [24] and the severity of disease on the Mayo’s Disease Activity Index [25]. Only patients with active burst (index >9) off immunosuppressive treatment were included. Lastly, not-treated and symptomatic BV infections defined by Nugent scoring of >7 and suspected of *Gardnerella vaginalis* origin were confirmed by microscopic examination (more than 20% of clue cells). Patients with other STI were excluded.

### Human T cell phenotype

Blood was collected and processed within 4 hours maximum. An ammonium chloride-based lysing reagent (BD Pharm Lyse, BD Biosciences) was used for erythrocyte deletion of 1ml of blood. After washing, cells were suspended in PBS and stained with Aqua Dye (Invitrogen) for cell viability. Cells were washed again, suspended in staining buffer and divided in four tubes. The four different panels assessed contained some common and some specific antibodies. Common antibodies were: CD3-eFluor 605, CD4-Alexa700 (eBioscience, San Diego, CA), CCR7-Horizon PE-CF594, CD38-Brillant Violet 421, HLA-DR-PerCP-Cy5.5 and CD11c-PE-Cy7 (BD Biosciences). Specific for each panel were: 1) CCR2-PE, CCR5-APC-Cy7, CXCR6-APC (R&D Systems Inc.) and CXCR3-FITC (BioLegend); 2) CD49d (α4)-FITC, β7-APC, CCR9-PE (BD Biosciences) and CD29 (β1)–APC-Cy7 (BioLegend); 3) CD103-FITC, CD54-APC, CD49a (α1)-PE and CD29–APC-Cy7 (BioLegend); 4) CD18-APC, CLA-FITC (BD Biosciences) and CCR10-PE (BioLegend). Cells were acquired using a BD LSRFortessa SORP flow cytometer (Flow Cytometry Platform, IGTP) and analyzed with FlowJo 9.3.2 software (TreeStar). Gates were drawn based on fluorescence minus one-controls and isotypes, and CD3^+^ CD4^-^ phenotype was considered CD8^+^ T cells.

### Tissue processing and flow cytometry

Mouse spleen and iliac lymph nodes were harvested and a single-cell suspension prepared. Peripheral blood was collected by retro-orbital bleeding. Red blood cells were removed by ACK lysing buffer (Life Technologies). Leukocytes were washed with FACS buffer (PBS with 2% FBS) and stored on ice until use. Mouse genital tract were removed and leukocytes isolated as described [26]. Briefly, mouse genital tract (vagina, cervix, uterine horns, oviducts) were minced into small pieces, digested in 500mg/L collagenase IV (Sigma) for 1 hour at 37°C with constant stirring. Leukocytes were purified by percoll density gradient centrifugation (GE Healthcare), washed with FACS buffer and stored on ice until use.

Single cell suspensions from spleen, dLNs, blood and genital tract were prepared in FACS buffer and blocked with Fc block (culture supernatant from the 24G2 hybridoma, 2% mouse serum, 2%, rat serum, and 0.01% sodium azide). Cells were then stained with anti-CD11b, F4/80 and B220-FITC together with MHCII PerCP-Cy5.5 (as dump channels), CD4-APC-eFluor780, CD8-Pacific Orange, CD44-Alexa 700 and CD11c-APC. All flow antibodies were obtained from eBiosciences. Samples were acquired on an LSRFortessa flow cytometer and analyzed using FlowJo software (TreeStar).

### Statistical Analysis

Data are reported as the median and IQR for each animal group or cohort using Prism 4.0 software (GraphPad Software). Statistical analyses were performed by non-parametric Mann Whitney test to compare single time points between two groups (case *vs*. control) using SPSS software for Windows version 13.0 (Chicago, IL). P value of <0.05 was considered significant.

### Accession codes

Microarray data presented in this article are deposited into the Gene Expression Omnibus (http://www.ncbi.nlm.nih.gov/geo/) under accession number GSE67147.

## Results

### Differentially expressed genes after genital tract infection in mice

**Fig 1:**
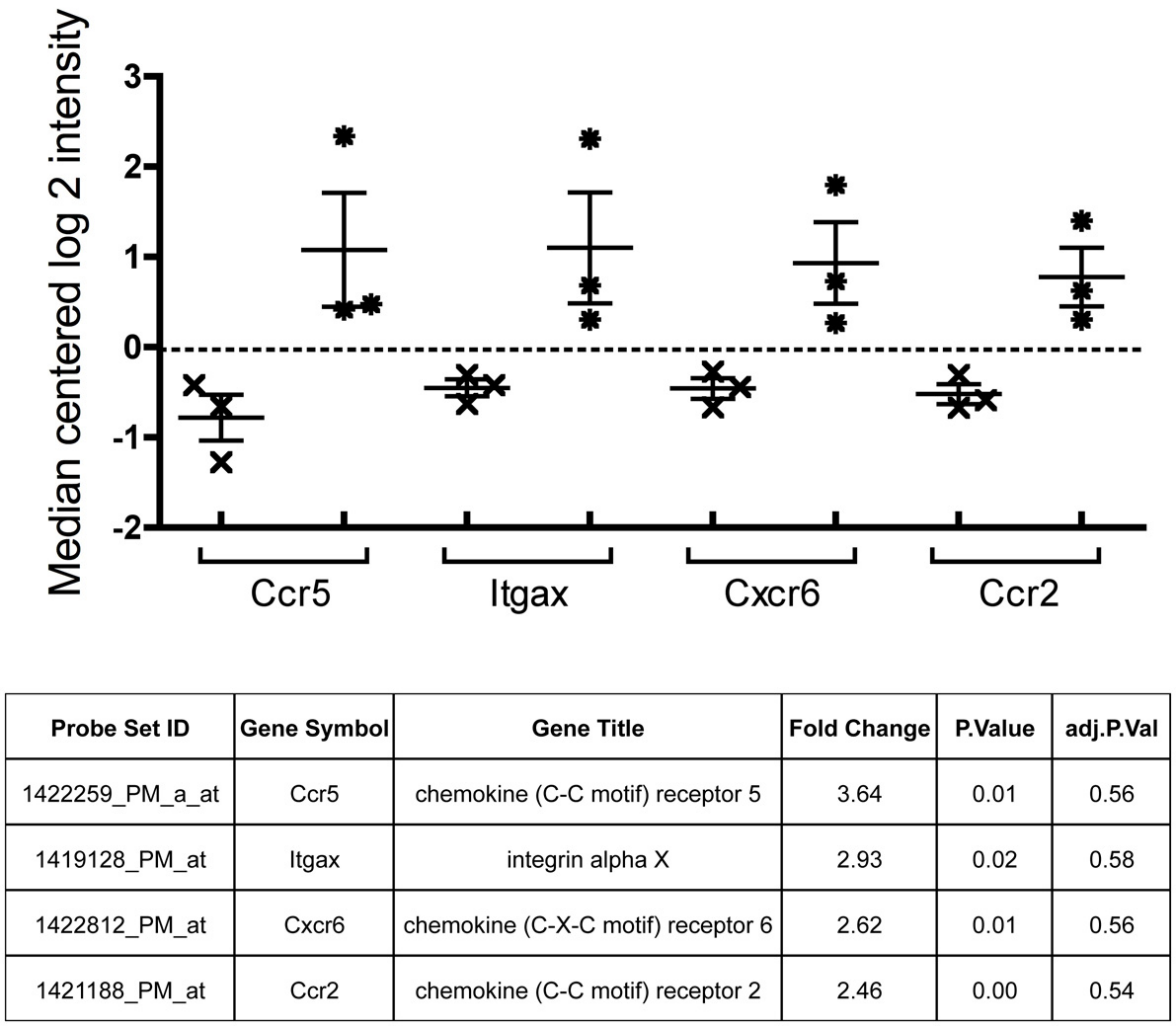
Adhesion molecule related genes overexpressed in circulating activated T_EM_ cells from vaginally *Chlamydia*-infected mice. Median centered log 2 intensity values derived from Affymetrix microarray hybridization experiments comparing non-infected control mice (n = 3, x symbol) vs. vaginally-infected mice (n = 3, _*_ symbol) for the *Ccr5*, *Itgax*, *Cxcr6* and *Ccr2* genes are shown. Fold change: Average fold change of vaginally-infected vs. control mice. P value: Nominal p-value. Adj p val: false discovery rate adjusted p-value.

To address the homing profile of T_EM_ cells shortly after vaginal infection, blood CD3^+^ CD62L^-^ CD44^+^ T cells were sorted from *Chlamydia*-infected mice (VAG group) and sham-treated mice (control group) 7 days post-infection in a pilot experiment. After qualitative assessment of the sample RNA integrity and amplification, we chose three samples in each group that qualified for microarray analyses. We detected highly significant differences in genes involved in interferon signaling, synthesis of DNA, cell cycle and activation of the immune system between the two groups (Table 1). Regarding adhesion molecules, several chemokine receptors and integrin genes were significantly up-regulated in infected animals compared to the control group and the four most significant genes were selected for further validation: *Ccr5*, *Itgax*, *Cxcr6* and *Ccr2* (Fig 1).

**Table 1:** Functions enriched among the top up-regulated genes in activated effector T cells from *Chlamydia*-infected vs. uninfected mouse samples with Canonical pathways.

Gene Set Name	# Genes in Gene Set (K)^a^	Description	# Genes in overlap (k)^b^	k/K^c^	FDR^d^ q-value
REACTOME_SYNTHESIS_OF_DNA	92	Genes involved in Synthesis of DNA	7	0.0761	7.02E-05
REACTOME_INTERFERON_SIGNALING	159	Genes involved in Interferon Signaling	8	0.0503	7.02E-05
REACTOME_ANTIVIRAL_MECHANISM_BY_IFN_STIM. GENES	66	Genes involved in Antiviral mechanism by IFN-stimulated genes	6	0.0909	7.02E-05
REACTOME_S_PHASE	109	Genes involved in S Phase	7	0.0642	7.02E-05
REACTOME_IMMUNE_SYSTEM	933	Genes involved in Immune System	16	0.0171	1.33E-04
PID_TCPTP_PATHWAY	43	Signaling events mediated by TCPTP	5	0.1163	1.33E-04
REACTOME_CELL_CYCLE	421	Genes involved in Cell Cycle	11	0.0261	1.33E-04
REACTOME_CYTOKINE_SIGNALING_ IN_IMMUNE_SYSTEM	270	Genes involved in Cytokine Signaling in Immune system	9	0.0333	1.69E-04
REACTOME_CELL_CYCLE_MITOTIC	325	Genes involved in Cell Cycle, Mitotic	9	0.0277	6.65E-04
REACTOME_DNA_STRAND_ ELONGATION	30	Genes involved in DNA strand elongation	4	0.1333	6.65E-04
REACTOME_ACTIVATION_OF_THE_ PRE_REPLICATIVE_COMPLEX	31	Genes involved in Activation of the pre-replicative complex	4	0.129	6.92E-04
REACTOME_DNA_REPLICATION	192	Genes involved in DNA Replication	7	0.0365	1.02E-03

^a^ K indicates the number of genes in the set from MSigDB.

^b^ k indicates the number of genes in the intersection of the query set with a set fromMSigDB.

^c^ k/K indicates the proportion of gene set genes present in the query set.

^d^ FDR corresponds to false discovery rate.

### Higher frequency of circulating CD11c^+^ CD8^+^ T cells is detected early after Chlamydia infection

**Fig 2:**
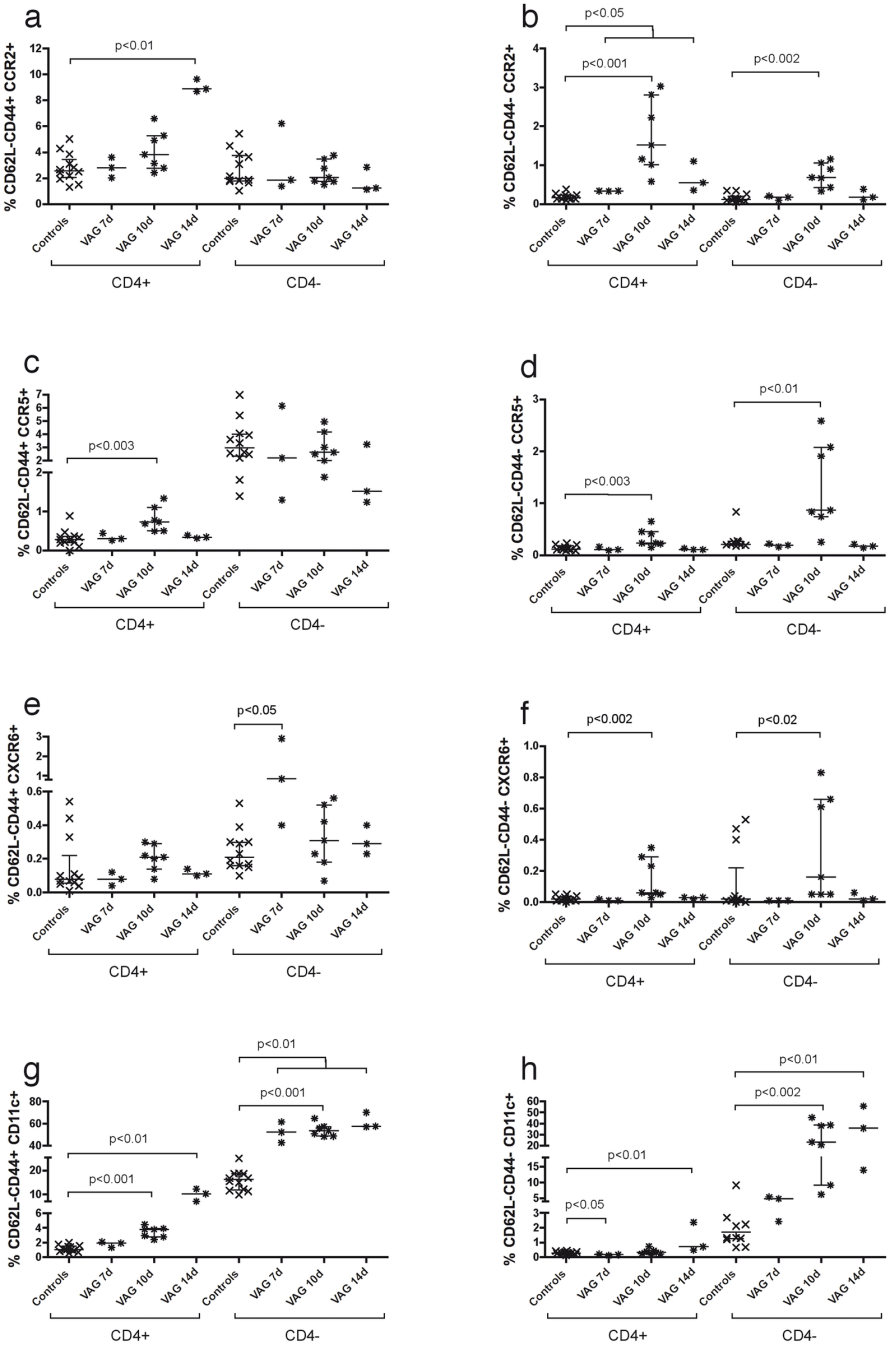
Kinetics of CCR2, CCR5, CXCR6 and CD11c frequency after vaginal *Chlamydia* infection in mice. The frequency of CCR5 (**a, b**), CCR2 (**c, d**), CXCR6 (**e, f**) and CD11c (**g, h**) was determined in activated CD44^+^ (left graphs) and CD44^-^ (right graphs) effector memory T (T_EM_) cells from blood by flow cytometry at 7, 10 and 14 days after vaginal infection with *C*. *muridarum* in mice. After gating on live CD3^+^ cells and CD4^+^ or CD4^-^ (putative CD8^+^) T cells, the frequency of CCR5, CCR2, CXCR6 and CD11c was quantified in the CD62L^-^ CD44^+^/CD44^-^ T cell subsets. Each time point represents the median ± interquartile range of three or seven infected animals and all controls (n = 12).

In order to confirm the expression of these molecules, we analyzed a 10-color panel by flow cytometry at 7, 10 and 14 days post-infection. In general, the percentage of CCR2, CCR5, CXCR6 and CD11c (*Itgax*) in live CD4^+^ or CD4^-^ (putative CD8^+^) T cells peaked 10 days after infection, when in most cases their total frequency was significantly higher than in the control group (S1 Fig). Of note, there was a significant increase in the frequency of T_EM_ cells (CD62L^-^) in the VAG groups compared to control animals. While CD4^+^ T cells only had a significant increase in the percentage of CD44^+^ activated T_EM_ cells (S1e Fig), CD8^+^ T cells demonstrated a significant increase in both, CD44^+^ and CD44^-^ subsets after infection (S1e and S1f Fig). When analyzing the expression of the selected homing molecules in the activated T_EM_ cell subset, there was a higher percentage of CCR5, CCR2 and CD11c in the CD4^+^ CD44^+^ T_EM_ cells at 10 and/or 14 days post-infection than in the control group (Fig 2a, 2g, 2e and 2c). In contrast, we detected a higher percentage of CXCR6 seven days after infection and of CD11c at all-time points in the CD8^+^ CD44^+^ T_EM_ cells compared to the control group (Fig 2e and 2g). Although expression of these markers was also increased in the non-activated fraction of both T_EM_ cell subsets, the magnitude of expression was much lower (Fig 2b, 2d, 2f and 2h). Thus, all the selected up-regulated genes were confirmed by protein expression. While CCR2 and CCR5 were only increased in the CD4^+^ T_EM_ cell subset after infection, expression of CXCR6 and CD11c was much more abundant in CD8^+^ T_EM_ cells.

### CD11c expression increases in human circulating CD8^+^ T_EM_ during symptomatic vaginosis

**Fig 3:**
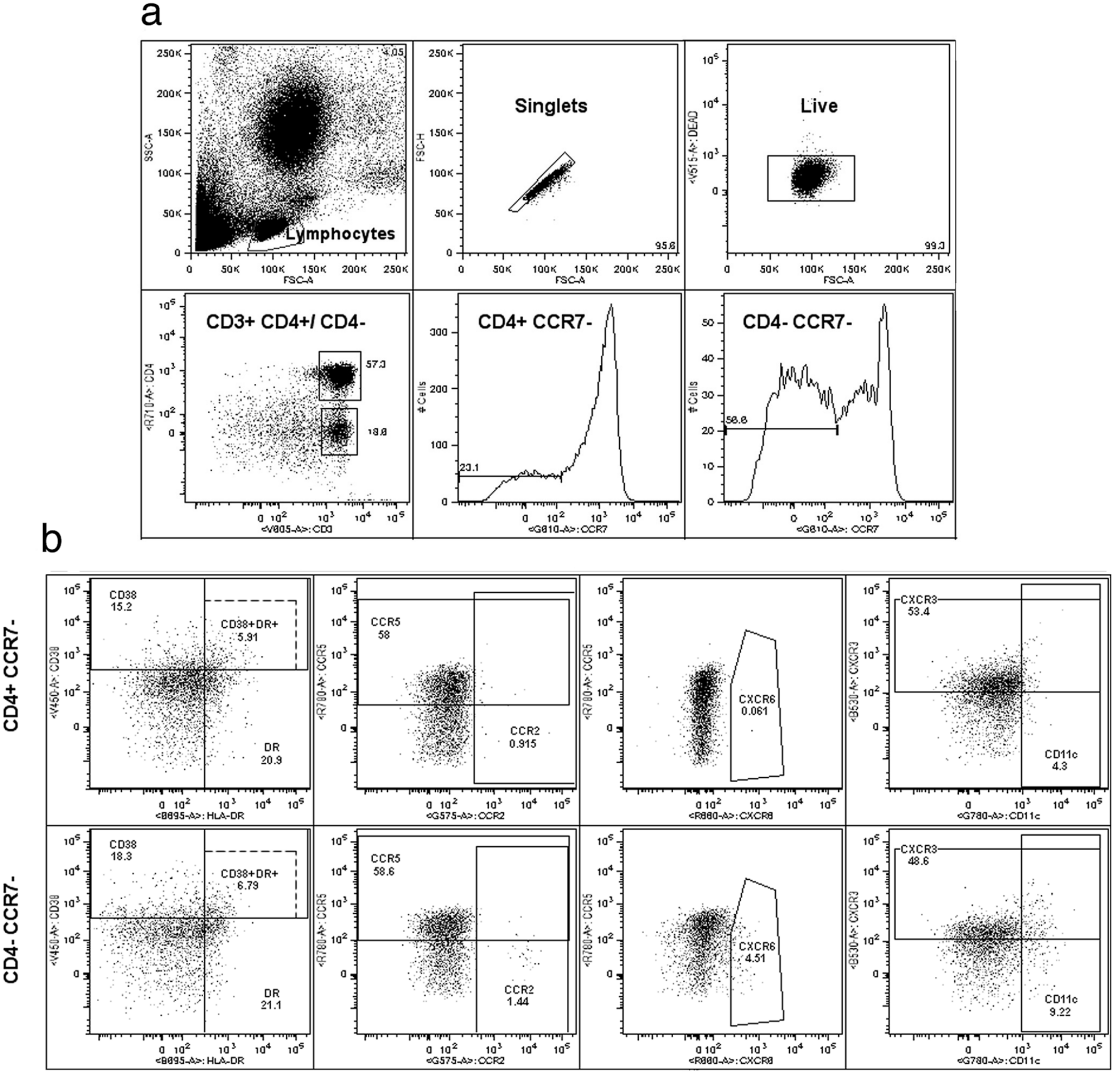
Gating strategy and representative plots of adhesion molecule analysis in circulating T_EM_ cells from women. The overall gating strategy for a representative single normal donor is shown. (**a**) General gating strategy for effector memory T (T_EM_) cells consist of the following consecutive gates: lymphocytes, singlets and live CD3^+^ T cells (top row); CD4^+^ and CD4^-^ (putative CD8^+^) T cells, and the effector CCR7^-^ fraction for each of these subsets (bottom row). (**b**) Representative plots of molecules analyzed in T_EM_ cells in one of the panels are shown: activated CD38 and/or HLA-DR T_EM_ cells, expression of CCR5 and CCR2, expression of CXCR6, and expression of CXCR3 and CD11c for CD4^+^ T_EM_ cells (top row) and CD8^+^ T_EM_ cells (bottom row). Isotype controls are shown in S3 Fig.

**Fig 4:**
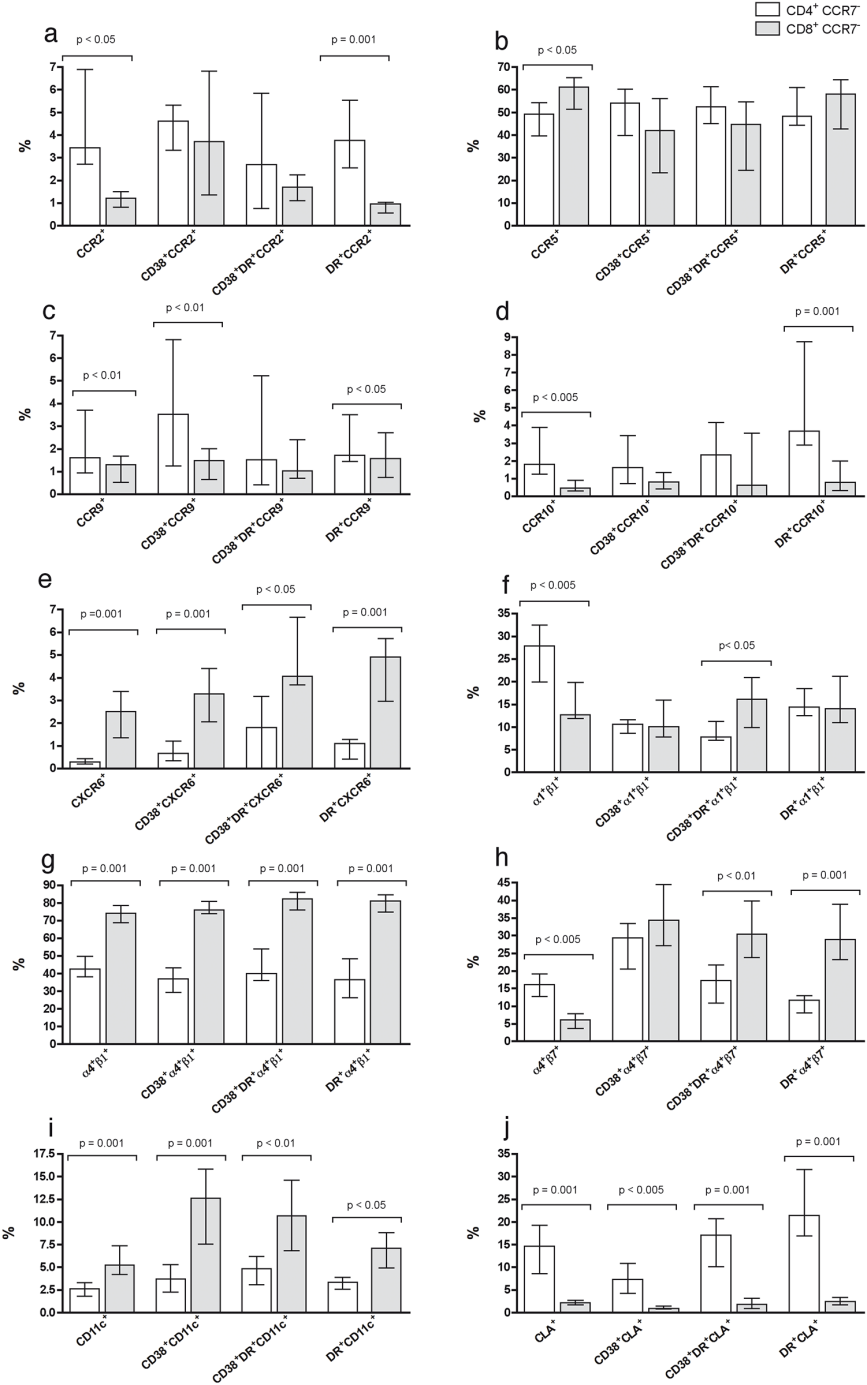
Comparison of adhesion molecule frequency in CD4 and CD8 T_EM_ cells from healthy women. A comparison between the frequency of (**a**) CCR5, (**b**) CCR2, (**c**) CCR9, (**d**) CCR10, (**e**) CXCR6, (**f**) α1β1, (**g**) α4β1, (**h**) α4β7, (**i**) CD11c and (**j**) CLA in CD4 (white bars) and CD8 (grey bars) effector memory T (T_EM_) cells was determined by flow cytometry. The frequency of each molecule was analyzed in total CD3^+^ T_EM_ cells and CD38^+^, CD38^+^ HLA-DR^+^ or HLA-DR^+^ activated fractions. General gating strategy is shown in Fig 3 and S2 Fig. Each bar represents the median ± interquartile range of the mean of healthy young women (n = 13).

We next addressed the expression of these and other potentially relevant mucosal homing molecules in peripheral blood T_EM_ cells from healthy young women (ND) in comparison to women with psoriasis (PS), ulcerative colitis (UC) and BV. First, we analyzed the expression of the different molecules by CD4^+^ and CD4^-^ (putative CD8^+^) T cells in CCR7^-^ T_EM_ cells from thirteen ND, as shown in the general gating strategy (Fig 3 and S2 Fig). All these molecules were also analyzed based on the activation markers included (CD38/HLA-DR) in order to detect associations between them. We observed a higher percentage of circulating CCR7^-^ CD4^+^ T cells expressing the CCR2, CCR9 and CCR10 chemokine receptors compared to CD8^+^ T cells, while there was a higher percentage of circulating CD8^+^ T_EM_ cells expressing CCR5 and CXCR6 compared to CD4^+^ T_EM_ cells (Fig 4). Regarding integrins and other molecules, the frequency of α1β1, α4β7 and CLA was in general higher in CD4^+^ T_EM_ cells than in CD8^+^, while the frequency of α4β1 and CD11c was higher in CD8^+^ T_EM_ cells (Fig 4). Finally, the frequency of CXCR3 did not show any differences between these subsets, while CD103 was only higher in the HLA-DR^+^ fraction of circulating CCR7^-^ CD4^+^ T cells compared to CD8^+^ T cells (S4 Fig).

**Fig 5:**
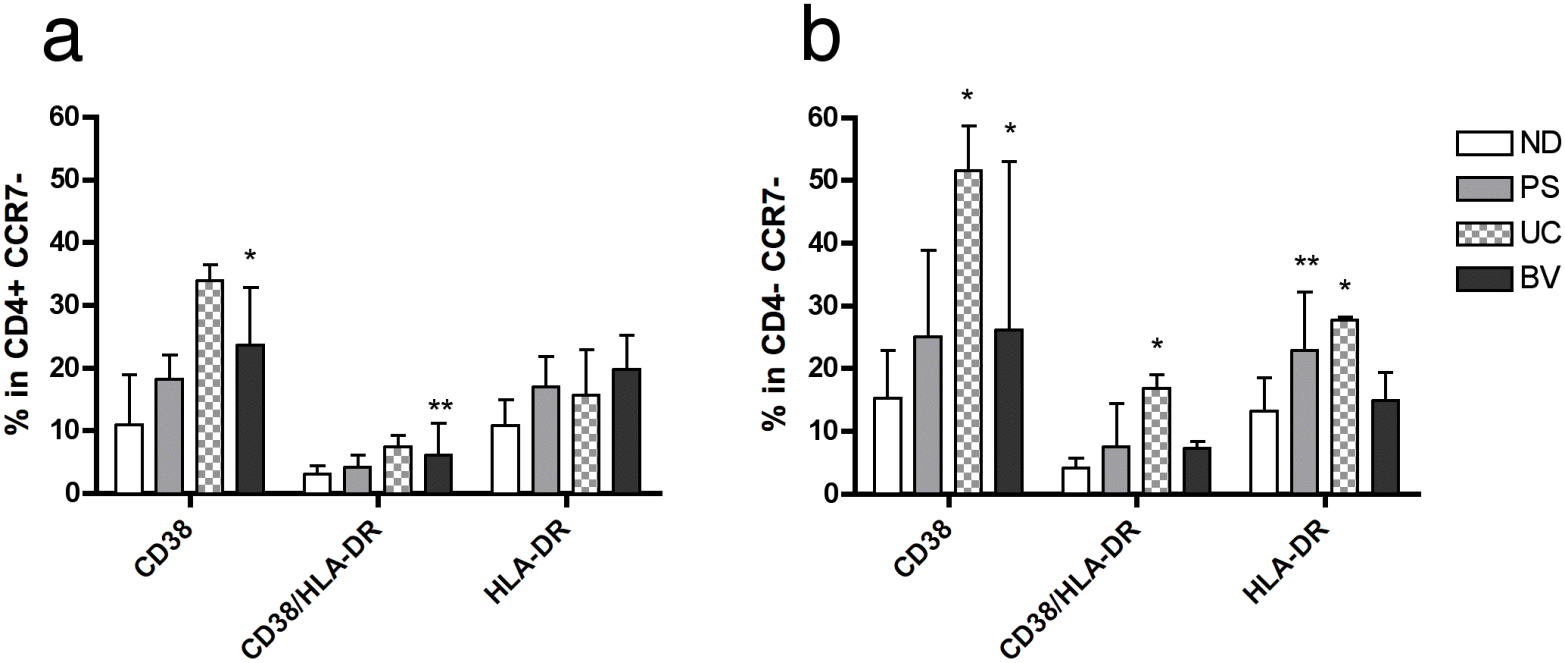
Comparison of activation markers frequency in T_EM_ cells from different conditions affecting women. The frequency of activated CD38^+^, CD38^+^ HLA-DR^+^ or HLA-DR^+^ CD4^+^ (**a**) and CD8^+^ (**b**) effector memory T (T_EM_) cells determined by flow cytometry is shown for normal donors (ND) and the different groups of patients. General gating strategy is shown in Fig 3 and S2 Fig. Each bar represents the median ± interquartile range of healthy young women (ND; white bars, n = 13), women with psoriasis (PS; grey bars, n = 5), ulcerative colitis (UC; checkered bars, n = 4) and bacterial vaginosis (BV; dark bars, n = 5). P values indicate: *<0.05; **<0.01.

Next we compared the expression of these adhesion molecules in ND with their expression in different mucosal and skin disorders. Of note, there was an overall decrease of the percentage of CD4^+^ T cells in the different groups of patients that was only significant for the BV cohort (S5 Fig). When analyzing the T_EM_ cell fractions, we detected a significant increase in at least one of the activation markers in each cohort, which denoted the pathological condition of the patients (Fig 5). The comparison on the expression of the different adhesion molecules was performed in the T_EM_ cell fraction as a total (S6 Fig), as well as in each of the activated fractions (S7 and S8 Figs). The main findings in total fractions were a general decrease on the frequency of CCR2 in CD4^+^ T_EM_ cells and an increase on the frequency of CCR10 in CD8^+^ T_EM_ cells from all groups of patients (S6 Fig). Also, there was a significant increase on the percentage of α1β1 expression in CD4^+^ T_EM_ cells from the PS group and a decrease on the percentage of CLA in the same cells compared to ND (S6c Fig). The UC group presented an increase on the percentage of α1β1 expression in CD8^+^ T_EM_ cells (S6d Fig), while the BV group had an increase on CCR5 and α4β1 expression in CD4^+^ T_EM_ cells and of CD11c in CD8^+^ T_EM_ cells when compared to ND (S6 Fig).

**Fig 6:**
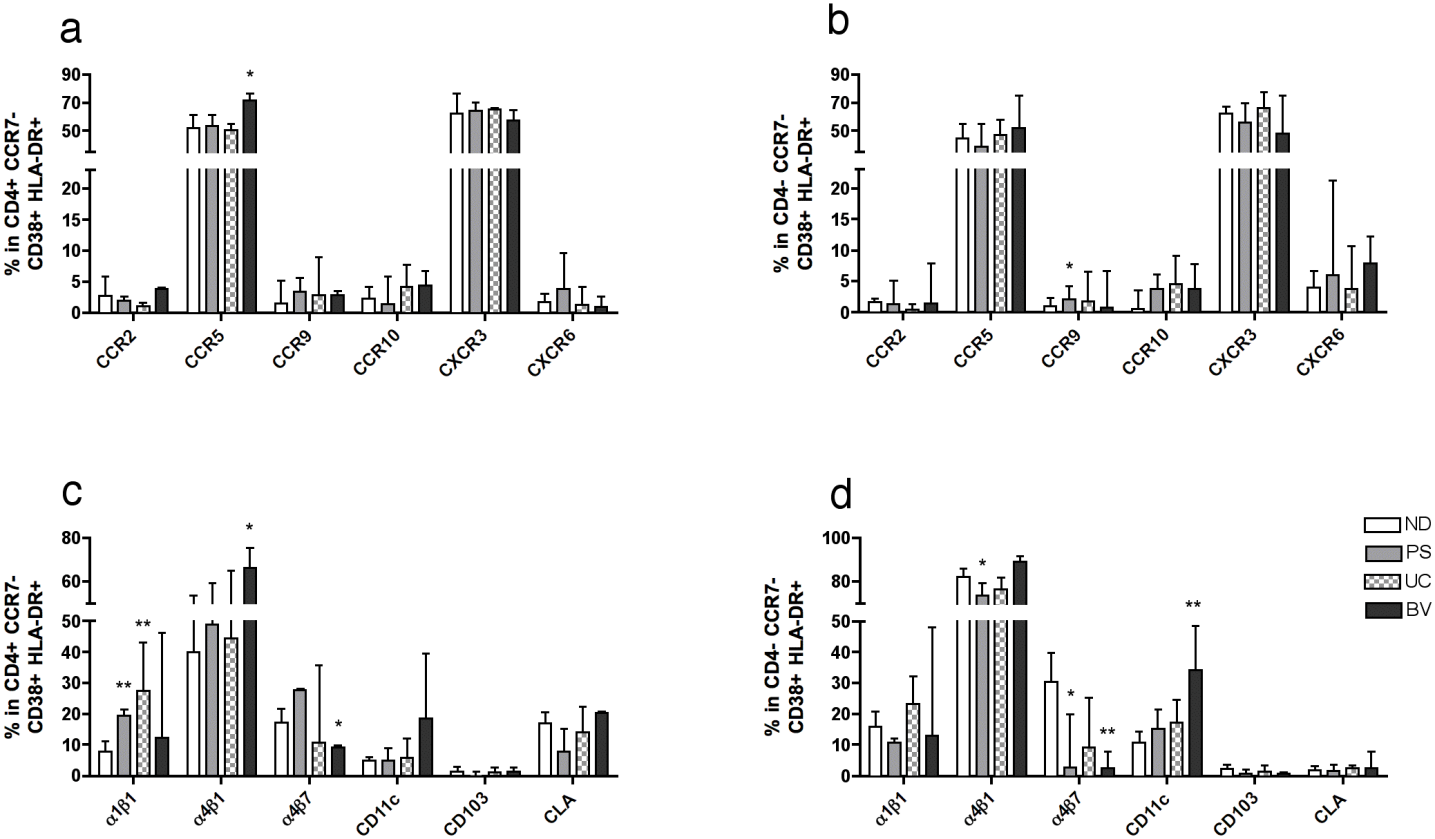
Frequency of adhesion molecules in CD38^+^ HLA-DR^+^ T_EM_ cells from different conditions affecting women. Percentages of the expression of chemokine receptors in CD38^+^ HLA-DR^+^ (**a**) CD4^+^ effector memory T (T_EM_) cells and (**b**) CD8^+^ T_EM_ cells determined by flow cytometry are shown for normal donors (ND) and the different groups of patients. Percentages of the expression of integrins and other adhesion molecules in CD38^+^ HLA-DR^+^ (**c**) CD4^+^ T_EM_ and (**d**) CD8^+^ T_EM_ cells determined by flow cytometry are shown for ND and the different groups of patients. General gating strategy is shown in Fig 3 and S2 Fig. Each bar represents the median ± interquartile range of healthy young women (ND; white bars, n = 13), women with psoriasis (PS; grey bars, n = 5), ulcerative colitis (UC; checkered bars, n = 4) and bacterial vaginosis (BV; dark bars, n = 5). P values indicate: *<0.05; **<0.01.

Since activated T_EM_ cells more likely contain specific T cells migrating towards the inflamed tissues [27], we focused on the differences detected in the double activated fraction (Fig 6). This way, the only significant changes within CD38^+^ HLA-DR^+^ T_EM_ cells from the PS group compared to ND were: increased frequency of CCR9 in CD8 T_EM_ cells, increased frequency of α1β1 in CD4^+^ T_EM_ cells, and a significant decrease of α4β1 and α4β7 in CD8^+^ T_EM_ cells (Fig 6). In fact, the decrease of α4β7 in CD8^+^ T_EM_ cells was a general observation in all groups of patients (Fig 6d), which was predominantly significant in the CD38^+^ fraction (S7 Fig). UC group characteristic profile in CD38^+^ HLA-DR^+^ T_EM_ cells was an increase of α1β1 percentage in CD4^+^ T_EM_ cells (as occurred in the PS group) (Fig 6c). Lastly, the BV group shared the decrease in the percentage of α4β7 expression in CD8^+^ T_EM_ cells with the PS group. As for the unique features in BV patients, we detected higher frequency of CCR5 and α4β1 in CD4^+^ T_EM_ cells with a concomitant decrease in the frequency of α4β7 in these cells, and also exclusive high percentage of CD11c in CD8^+^ T_EM_ cells (Fig 6). In summary, increased expression of CCR5 and CD11c on CD4^+^ and CD8^+^ T_EM_ cells respectively was confirmed in women with symptomatic BV.

### CD11c expression in blood after vaginal infection correlates with an increase in the genital tract but is not exclusive of infection in these tissues

**Fig 7:**
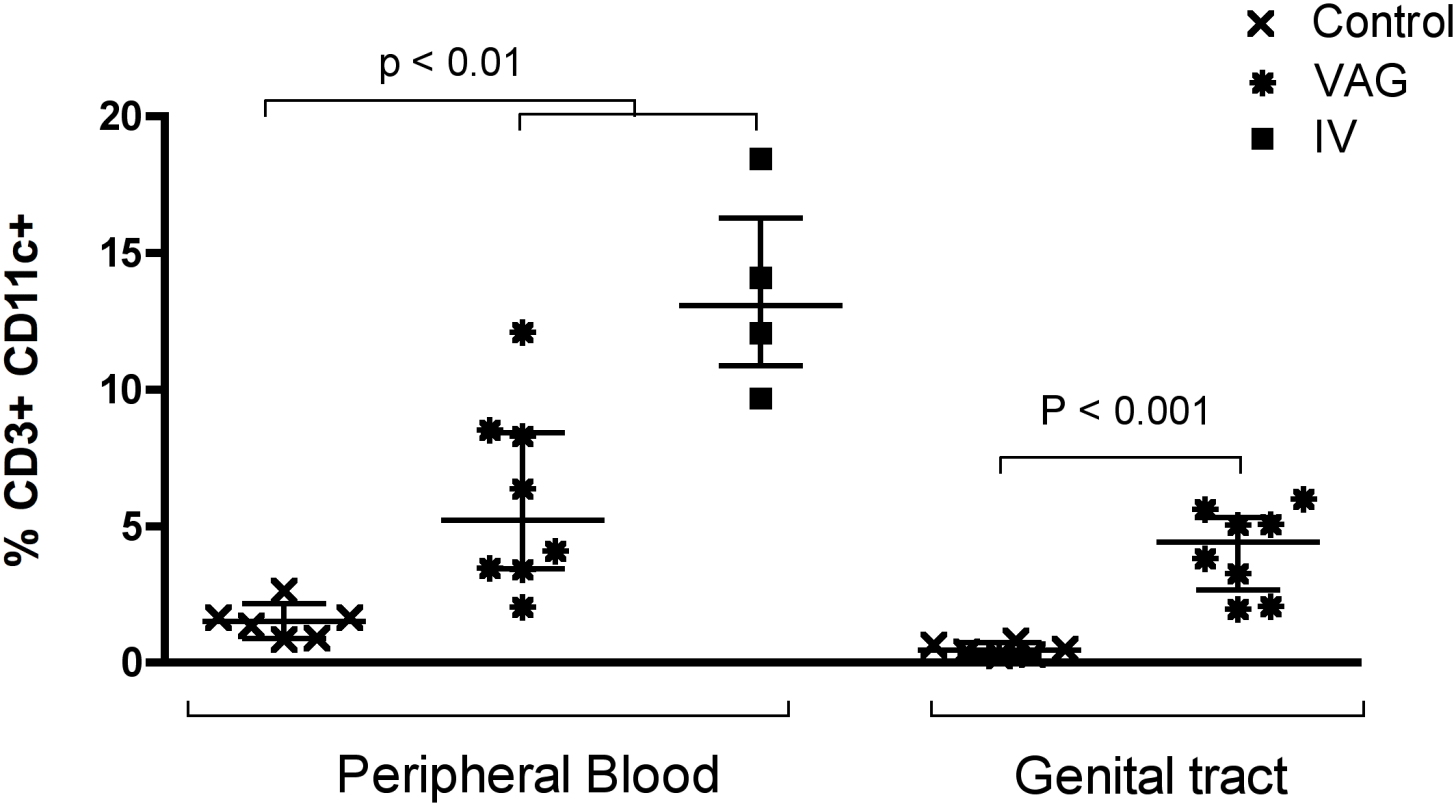
CD11c expression in T cells from blood and genital tract after vaginal or systemic *Chlamydia* infection. The frequency of CD11c in CD3^+^ T cells was determined by flow cytometry 7 days after vaginal (VAG) or intravenous (IV) infection with *C*. *muridarum* in blood or genital tract from mice. Each time point represents the median ± interquartile range of controls (n = 6), VAG-infected animals (n = 8) and IV-infected animals (n = 4).

**Fig 8:**
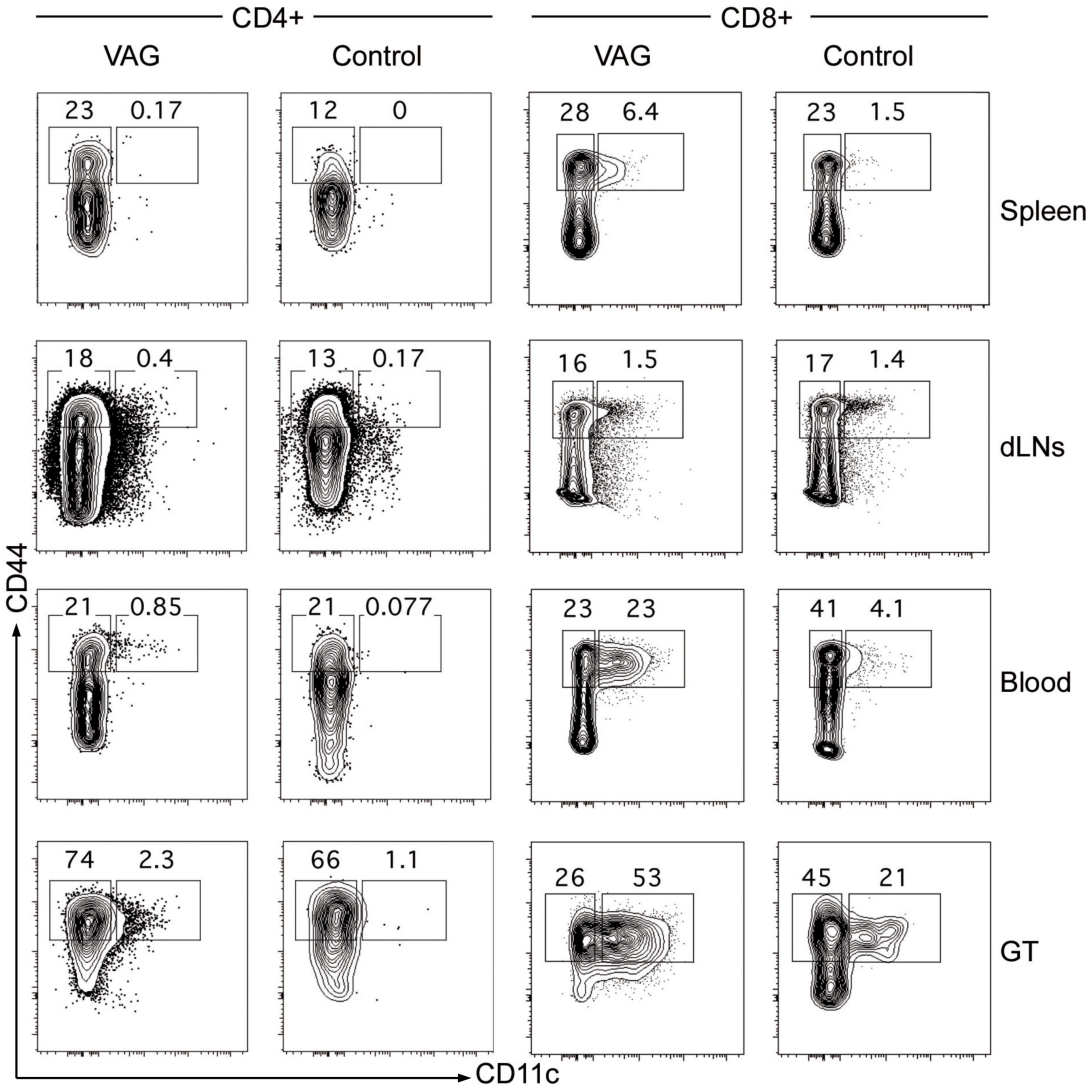
CD11c expression in activated CD4 /CD8 cells from different tissues after vaginal *Chlamydia* infection in mice. The frequency of CD44^+^ CD11c^+^ in CD4^+^ (left panels) and CD8^+^ cells (right panels) was determined by flow cytometry 14 days after vaginal (VAG) infection with *C*. *muridarum* in spleen, draining lymph nodes (dLNs), blood and genital tract (GT) from mice. Examples from one infected and one control animal are shown.

Considering that CD11c was the most striking and novel marker of genital tract condition in both, mice and women, we performed additional experiments to address if this increase was exclusive of productive infection in these tissues or, in contrast, was a consequence of bacterial infection in general. Therefore, we performed a new set of animal experiments in which we included an intravenously (IV) *Chlamydia*-infected group. It is important to note that IV infection induces bacterial replication in different systemic and also mucosal tissues, including spleen and lung [21]. Interestingly, seven days post-infection the frequency of CD11c^+^ cells on blood T cells was much higher in the IV group (median: 13.1% [IQR: 10.9–16.3]) than in any other group (median: 1.51%, [IQR: 0.9–2.2] in the controls or median: 5.23% [IQR: 3.4–8.4] in the VAG group) (Fig 7). In the control and the VAG groups we obtained cell suspensions from the genital tract to determine the frequency of CD11c^+^ T cells. As shown (Fig 7), seven days after infection, the total frequency of CD3^+^ CD11c^+^ in genital tract increased from a median of 0.46% [IQR: 0.28–0.74] in the control group to a median of 4.43% [IQR: 2.68–5.33] in the VAG group. Additional assessment of the expansion of CD11c^+^ CD44^+^ cells in different tissues, including spleen, draining lymph nodes (dLNs), blood and genital tract of the VAG infected animals 14 days post-infection demonstrated that most of these cells are CD8^+^ that expand in spleen, blood and, mainly, in the genital tract (Fig 8). This way, while CD11c^+^ CD44^+^ represented 0.37% [IQR: 0.47–0.85] of the CD4^+^ cells in spleen, 0.61% [IQR: 0.40–0.82] in dLNs, 0.83% [IQR: 0.17–0.41] in blood and 4.2% [IQR: 2.3–4.7] in genital tract of the VAG-infected mice, these CD11c^+^ CD44^+^ cells were more frequent in the CD8^+^ cell fraction from all these tissues: 6.4% [IQR: 6.3–10] in the spleen, 1.9% [IQR: 1.5–2.1] in the dLNs, 23% [IQR: 15–42] in blood and 53% [IQR: 46–53] in the genital tract. Thus, 7 and 14 days after infection, CD11c expression increases in the genital tract of mice correlating with the increase observed in blood. However, CD11c increased expression is not specific to infection in the genital tract, since systemic infection also expands this subset.

## Discussion

We interrogated the expression of multiple adhesion molecules in circulating T_EM_ cells from different groups of women with disorders affecting primarily peripheral tissues. We report for the first time that women with BV have higher frequency of CD8^+^ T_EM_ expressing CD11c. This increase was not observed during inflammatory pathologies affecting the gastrointestinal tract or the skin. Importantly, we also detected an increase in CD11c gene and protein expression in a mouse model of *Chlamydia* reproductive tract infection in both, blood and genital tract. Other patterns of integrin and chemokine receptor expression were also observed in patients with BV. In particular, the expression of CCR5 and α4β1 was increased in circulating CD4^+^ T_EM_ cells of these women compared to healthy donors.

The increased expression of CD11c in T_EM_ cell subsets soon after vaginal infection or during an episode of vaginosis was a strong and consistent finding in mice and women. Although the expression of CD11c has been classically used to identify dendritic cells, this α-chain integrin is also expressed in other myeloid cells, NK cells and populations of activated T and B cells [28]. Specifically within the activated T cell fraction, CD11c expression has been associated to effector and regulatory T cells in different mouse models [29–33], as well as with intraephitelial lymphocytes in the small gut [34, 35]. More recently, some authors have described the existence of a unique subset in mice and importantly, in humans, that combine key features of T and dendritic cells [36]. These cells are positive for T cell receptor, major histocompatibility complex II and CD11c^+^ and, regardless of their lymphocyte morphology, posses antigen presenting cell and innate-like properties [36]. However, to our knowledge, no specific report on expression of CD11c in human T cells in association to a mucosal condition has ever been published.

A considerable constraint in our study is the comparison between the cohorts evaluated. Although all these disorders have a clear Th1 component [37–39], they have very different origins and associated inflammation, and no other mucosal disorder with a bacterial component could be compared to the BV group. This fact, the *Chlamydia* systemic infection experiment results (Fig 7) and supporting animal models that demonstrate CD11c expression in T cells after systemic vaccination and infection [29–33], indicates that CD11c expression is not specific to genital tract infection, rather indicative of activation and recirculation in general. Moreover, there was a trend towards higher percentage of CD11c^+^ in T cells from patients with UC that was significant in the HLA-DR fraction (S8 Fig). Although we acknowledge the limitation of the sample size for the cohorts included in the study, the present results represent a unique comparison between peripheral tissue alterations that provide adhesion molecules of interest for each of the different disorders to further explore. In any case, this is the first report demonstrating enhanced CD11c expression in CD8^+^ T_EM_ cells after intravaginal *Chlamydia* infection in mice and symptomatic BV in women, and we are currently working on defining the phenotype and role of these cells in these models.

The differential expression of certain adhesion molecules can induce a site-dependent homing profile that may work together with some other generic signals (such as CXCR3, CCR3, CCR5 and CCR6) [10]. Previous studies have examined adhesion molecules necessary for homing to the upper genital tract in a mouse model of *C*. *trachomatis* infection by directly infecting the uterine horns [14, 40]. In this model, CXCR3, CCR5 and α4β1 are required for homing of protective T cells to the murine upper genital tract [14, 40]. Interestingly, samples from women with symptomatic BV, confirmed two of these molecules as potentially involved in FGT homing in humans. The frequency of integrin α4β1 was indeed up regulated in CD4^+^ T_EM_ cells, which correlates with high expression of this integrin in the genital mucosa of women [41]. Moreover, BV patients did also showed a higher percentage of CCR5 expression in their CD4^+^ T_EM_ cells compared to ND, indicating that CCR5 might indeed be necessary for FGT homing as suggested [40]. Asymptomatic genital HSV-2 infection is also associated with increased expression of CCR5 by endocervical CD4^+^ T cells and similar trends were observed in circulating CD4^+^ T cells [18]. Further, a live-attenuated vaccine that continuously replicates in systemic and mucosal tissues in macaques also induces increased frequency of peripheral CD4^+^ T cells expressing CCR5, and the two animals with the highest percentage of this population had the highest number of specific T cells in the genital tract [42]. However, a recent report on the effect of vaginal immunization in women showed a clear down-regulation of CCR5 on CD4^+^ T cells after immunization [43]. Nevertheless, the first time-point analyzed in that study was 4 weeks post-immunization, when major recirculation and infiltration of T cells may have already occurred [12]. Of great importance is the fact that this increment is detected in the CD4^+^ T_EM_ cell population, since CCR5 is a co-receptor for HIV and thus could have highly detrimental effects favoring HIV infection susceptibility [44, 45]. If higher numbers of CCR5 expressing CD4^+^ T_EM_ cells are indeed infiltrated in the genital tissue during BV episodes, this could partially explain its association with higher risk of HIV-1 acquisition [46].

Finally, while CLA expression in total CD4^+^ T_EM_ cells of the PS group was unexpectedly reduced compared to ND (S1c Fig), we detected higher frequency of CLA on CD8^+^ HLA-DR^+^ T_EM_ cells from the BV group (S8d Fig). Due to their migration to skin, the number of CLA^+^ T cells in the periphery decreases inversely to disease severity during acute psoriasis [47]. Further, CLA interacts with E-selectin expressed on venular endothelial cells not only from inflamed skin, but also from oral mucosa and FGT, and genital HSV-specific CD8^+^ T cells in the peripheral blood express high levels of CLA [16, 19]. Yet α4β7, another molecule described in asymptomatic HSV-2-infected patients but not in uninfected patients [18], was down regulated in activated T_EM_ cells from BV patients (Fig 6). While this decrease could also indicate selective infiltration of these cells into the infected tissues early after infection, the existence of a mechanism that would actually down-regulate some of these molecules cannot be discarded.

Many properties that enable T cells to traffic to specific locations are programmed during the early stages of the infection [11]. Thus, in order to understand the homing patterns and dynamics of the mucosal response associated to vaccination and infection, we need to analyze specific and activated T cells responses early after activation [4, 12]. In summary, in this study we define adhesion molecules, namely CCR5, α4β1 and CD11c, which may be desirable to induce in order to generate an effective mucosal response in vaccine candidates against STI. Special attention should be given to CD11c as a novel marker of T cell mucosal immunity in response to genital tract disorders.

## Supporting Information

S1 Fig Kinetics of the frequency of adhesion molecules and effector T cells after vaginal infection in mice. The frequency of CCR5 (**a**), CCR2 (**b**), CXCR6 (**c**), CD11c (**d**), CD62L^-^ CD44^+^ (**e**) and CD62L^-^ CD44^-^ (**f**) was determined in T cells from blood by flow cytometry at 7, 10 and 14 days after vaginal infection with *C*. *muridarum* in mice. After gating on live CD3^+^ cells and CD4^+^ or CD4^-^ (putative CD8^+^) T cells, the frequency of CCR5, CCR2, CXCR6, CD11c, CD62L^-^ CD44^+^ and CD62L^-^ CD44^-^ was quantified. Each time point represents the median ± interquartile range of three or seven infected animals and all controls (n = 12).
(TIF)

S2 Fig Representative plots of adhesion molecule analysis in circulating T_EM_ cells from women. The overall gating strategy for a representative single normal donor is shown in Fig 3. Representative plots of molecules analyzed in T_EM_ cells in each of the panels are shown for CD4^+^ T_EM_ cells (top row) and CD8^+^ T_EM_ cells (bottom row): (**a**) expression of CCR9, α4 and β7;(**b**) expression of α1, β1 and CD103 and (**c**) expression of CCR10 and CLA. Isotype controls are shown in S3 Fig.
(TIF)

S3 Fig Isotype controls for the molecules analyzed in circulating CD4^-^ T_EM_ cells from women. The cut-off determined by the isotype control for each adhesion or activation molecule analyzed is shown in zebra plots for the CD4^-^ CCR7^-^ T cells.
(TIF)

S4 Fig Comparison of CXCR3 and CD103 frequencies in CD4 and CD8 T_EM_ cells from healthy women. A comparison between the frequency of (**a**) CXCR3 and (**b**) CD103 in CD4 (white bars) and CD8 (grey bars) effector memory T (T_EM_) cells was determined by flow cytometry. The frequency of each molecule was analyzed in total CD3^+^ T_EM_ cells and CD38^+^, CD38^+^ HLA-DR^+^ or HLA-DR^+^ activated fractions. General gating strategy is shown in Fig 3 and S2 Fig. Each bar represents the median ± interquartile range of healthy young women (n = 13).
(TIF)

S5 Fig Frequency of CD4^+^ T cells during different conditions affecting peripheral tissues in women. The percentage of CD4^+^ T cells determined by flow cytometry is shown for ND and the different groups of patients. General gating strategy is shown in Fig 3. Each bar represents the median ± interquartile range of healthy young women (ND; white bars, n = 13), women with psoriasis (PS; grey bars, n = 5), ulcerative colitis (UC; checkered bars, n = 4) and bacterial vaginosis (BV; dark bars, n = 5). P value indicates: *<0.05.
(TIF)

S6 Fig Frequency of adhesion molecules in T_EM_ cells from different conditions affecting women. Percentages of the expression of chemokine receptors in total (**a**) CD4^+^ effector memory T (T_EM_) cells and (**b**) CD8^+^ T_EM_ cells determined by flow cytometry are shown for normal donors (ND) and the different groups of patients. Percentages of the expression of integrins and other adhesion molecules in total (**c**) CD4^+^ T_EM_ and (d) CD8^+^ T_EM_ cells determined by flow cytometry is shown for ND and the different groups of patients. General gating strategy is shown in Fig 3 and S2 Fig. Each bar represents the median ± interquartile range of healthy young women (ND; white bars, n = 13), women with psoriasis (PS; grey bars, n = 5), ulcerative colitis (UC; checkered bars, n = 4) and bacterial vaginosis (BV; dark bars, n = 5). P values indicate: *<0.05; **<0.01; ***<0.001.
(TIF)

S7 Fig Frequency of adhesion molecules in CD38^+^ T_EM_ cells from different conditions affecting women. Percentages of the expression of chemokine receptors in CD38^+^ (**a**) CD4^+^ effector memory T (T_EM_) cells and (**b**) CD8^+^ T_EM_ cells determined by flow cytometry are shown for normal donors (ND) and the different groups of patients. Percentages of the expression of integrins and other adhesion molecules in CD38^+^ (**c**) CD4^+^ T_EM_ and (d) CD8^+^ T_EM_ cells determined by flow cytometry are shown for ND and the different groups of patients. General gating strategy is shown in Fig 3 and S2 Fig. Each bar represents the median ± interquartile range of healthy young women (ND; white bars, n = 13), women with psoriasis (PS; grey bars, n = 5), ulcerative colitis (UC; checkered bars, n = 4) and bacterial vaginosis (BV; dark bars, n = 5). P values indicate: *<0.05; **<0.01; ***<0.001.
(TIF)

S8 Fig Frequency of adhesion molecules in HLA-DR^+^ T_EM_ cells from different conditions affecting women. Percentages of the expression of chemokine receptors in HLA-DR^+^ (**a**) CD4^+^ effector memory T (T_EM_) cells and (**b**) CD8^+^ T_EM_ cells determined by flow cytometry are shown for normal donors (ND) and the different groups of patients. Percentages of the expression of integrins and other adhesion molecules in HLA-DR^+^ (**c**) CD4^+^ T_EM_ and (d) CD8^+^ T_EM_ cells determined by flow cytometry are shown for ND and the different groups of patients. General gating strategy is shown in Fig 3 and S2 Fig. Each bar represents the median ± interquartile range of healthy young women (ND; white bars, n = 13), women with psoriasis (PS; grey bars, n = 5), ulcerative colitis (UC; checkered bars, n = 4) and bacterial vaginosis (BV; dark bars, n = 5). P values indicate: *<0.05; **<0.01; ***<0.001.
(TIF)
