# Supplementary figures and images for: Adhesion Molecules Associated with Female Genital Tract Infection
Source: PLoS One. 2016 Jun 7;11(6):e0156605. doi: 10.1371/journal.pone.0156605 (PMC4896633; doi:10.1371/journal.pone.0156605)

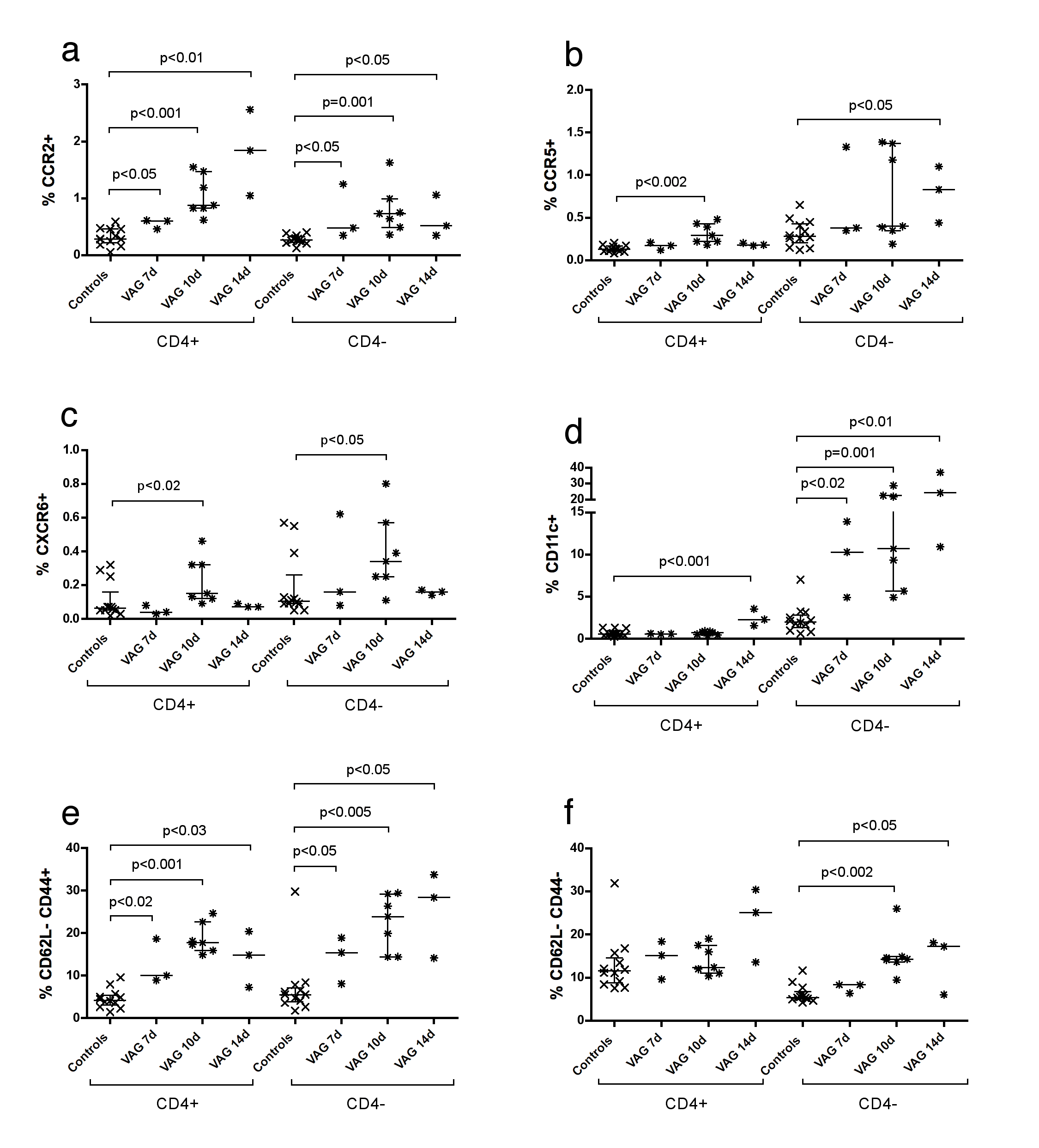

Supplement: S1 Fig — The frequency of CCR5 (a), CCR2 (b), CXCR6 (c), CD11c (d), CD62L- CD44+ (e) and CD62L- CD44- (f) was determined in T cells from blood by flow cytometry at 7, 10 and 14 days after vaginal infection with C. muridarum in mice. After gating on live CD3+ cells and CD4+ or CD4- (putative CD8+) T cells, the frequency of CCR5, CCR2, CXCR6, CD11c, CD62L- CD44+ and CD62L- CD44- was quantified. Each time point represents the median ± interquartile range of three or seven infected animals and all controls (n = 12). (TIF) [file pone.0156605.s001.tif]

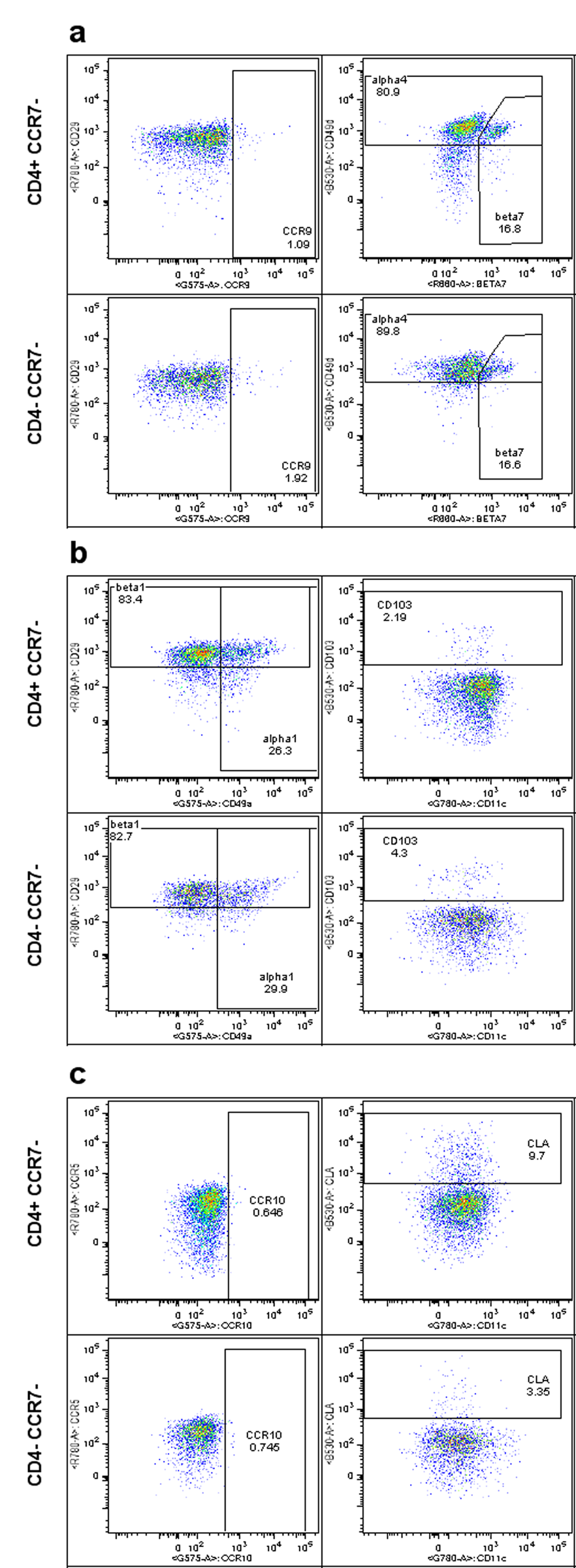

Supplement: S2 Fig — The overall gating strategy for a representative single normal donor is shown in Fig 3. Representative plots of molecules analyzed in TEM cells in each of the panels are shown for CD4+ TEM cells (top row) and CD8+ TEM cells (bottom row): (a) expression of CCR9, α4 and β7;(b) expression of α1, β1 and CD103 and (c) expression of CCR10 and CLA. Isotype controls are shown in S3 Fig. (TIF) [file pone.0156605.s002.tif]

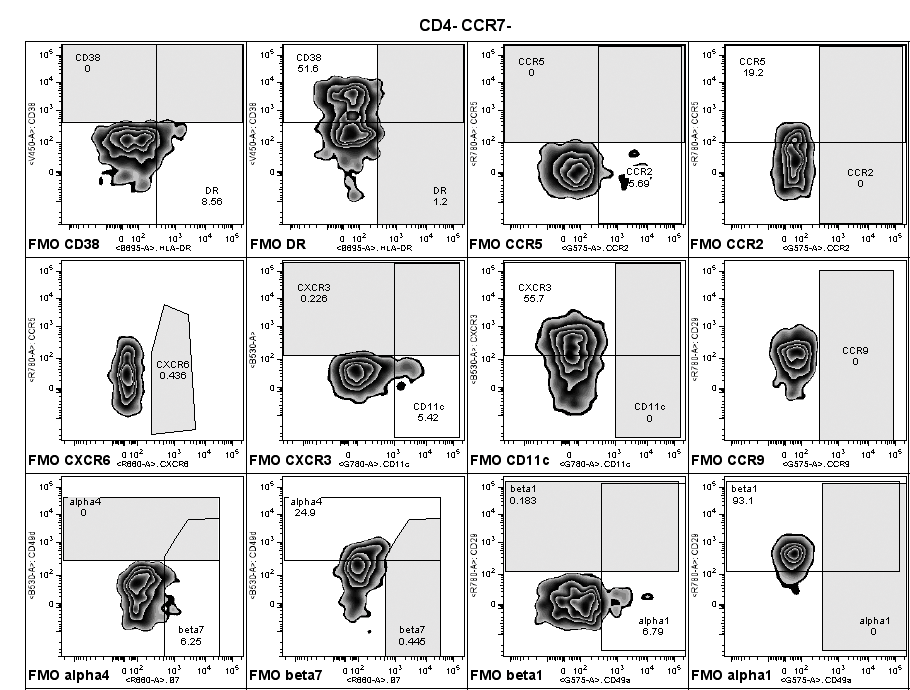

Supplement: S3 Fig — The cut-off determined by the isotype control for each adhesion or activation molecule analyzed is shown in zebra plots for the CD4- CCR7- T cells. (TIF) [file pone.0156605.s003.tif]

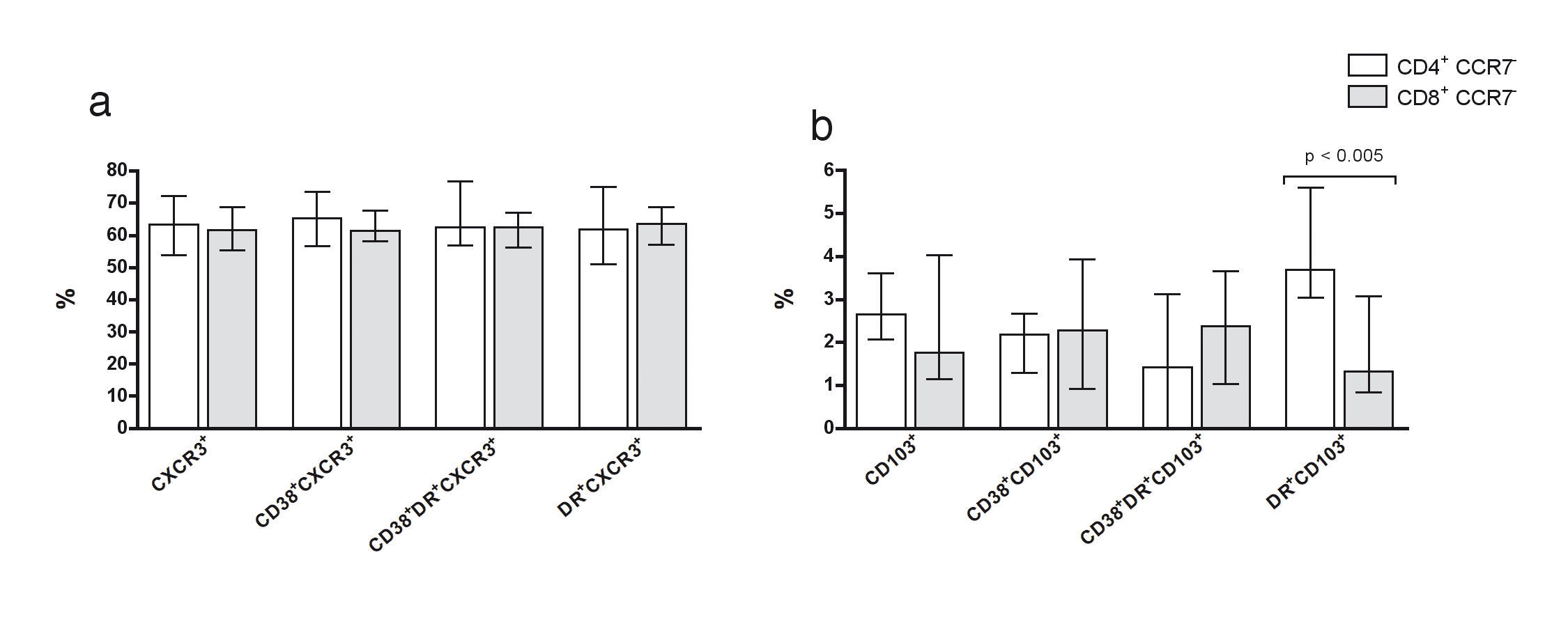

Supplement: S4 Fig — A comparison between the frequency of (a) CXCR3 and (b) CD103 in CD4 (white bars) and CD8 (grey bars) effector memory T (TEM) cells was determined by flow cytometry. The frequency of each molecule was analyzed in total CD3+ TEM cells and CD38+, CD38+ HLA-DR+ or HLA-DR+ activated fractions. General gating strategy is shown in Fig 3 and S2 Fig. Each bar represents the median ± interquartile range of healthy young women (n = 13). (TIF) [file pone.0156605.s004.tif]

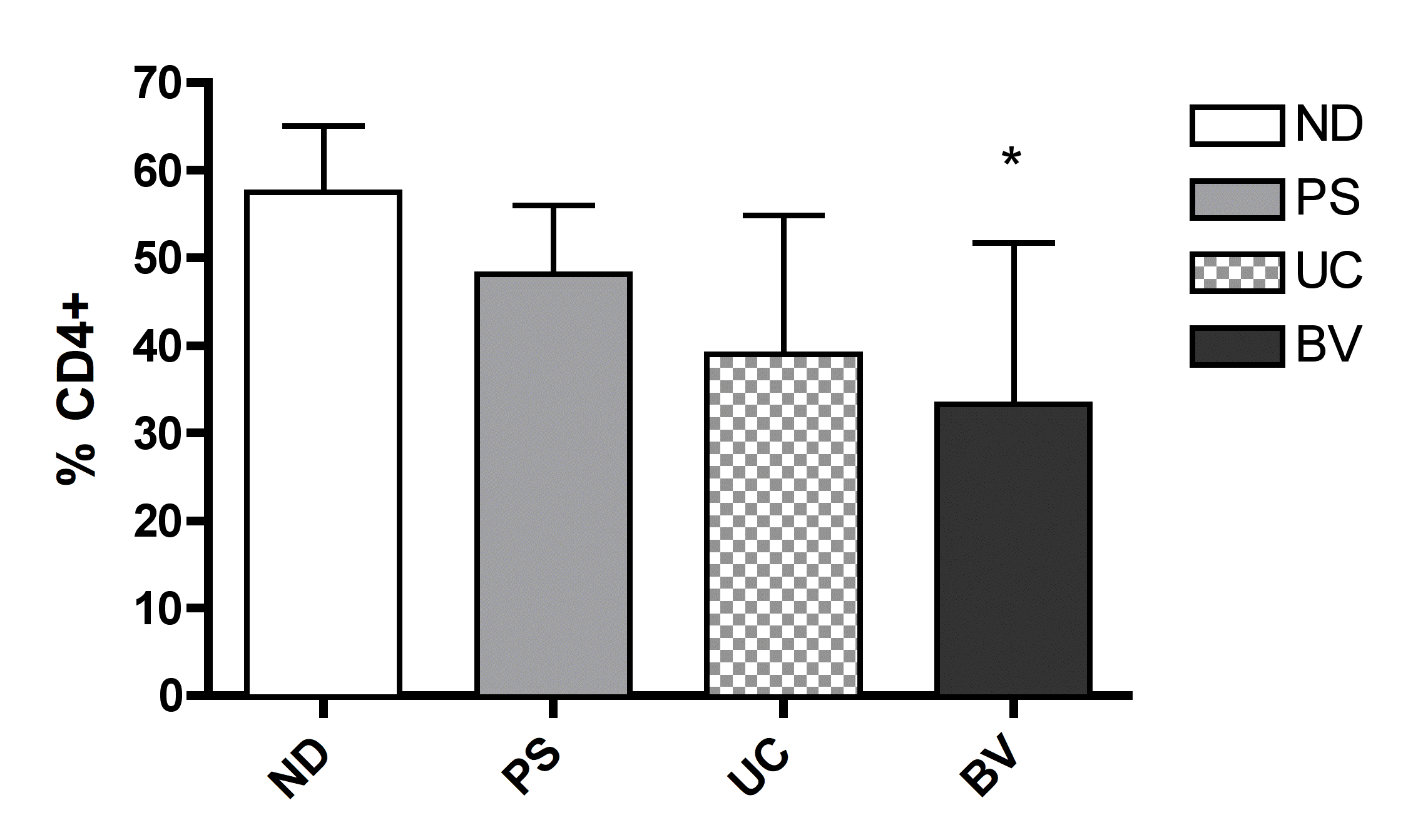

Supplement: S5 Fig — The percentage of CD4+ T cells determined by flow cytometry is shown for ND and the different groups of patients. General gating strategy is shown in Fig 3. Each bar represents the median ± interquartile range of healthy young women (ND; white bars, n = 13), women with psoriasis (PS; grey bars, n = 5), ulcerative colitis (UC; checkered bars, n = 4) and bacterial vaginosis (BV; dark bars, n = 5). P value indicates: *<0.05. (TIF) [file pone.0156605.s005.tif]

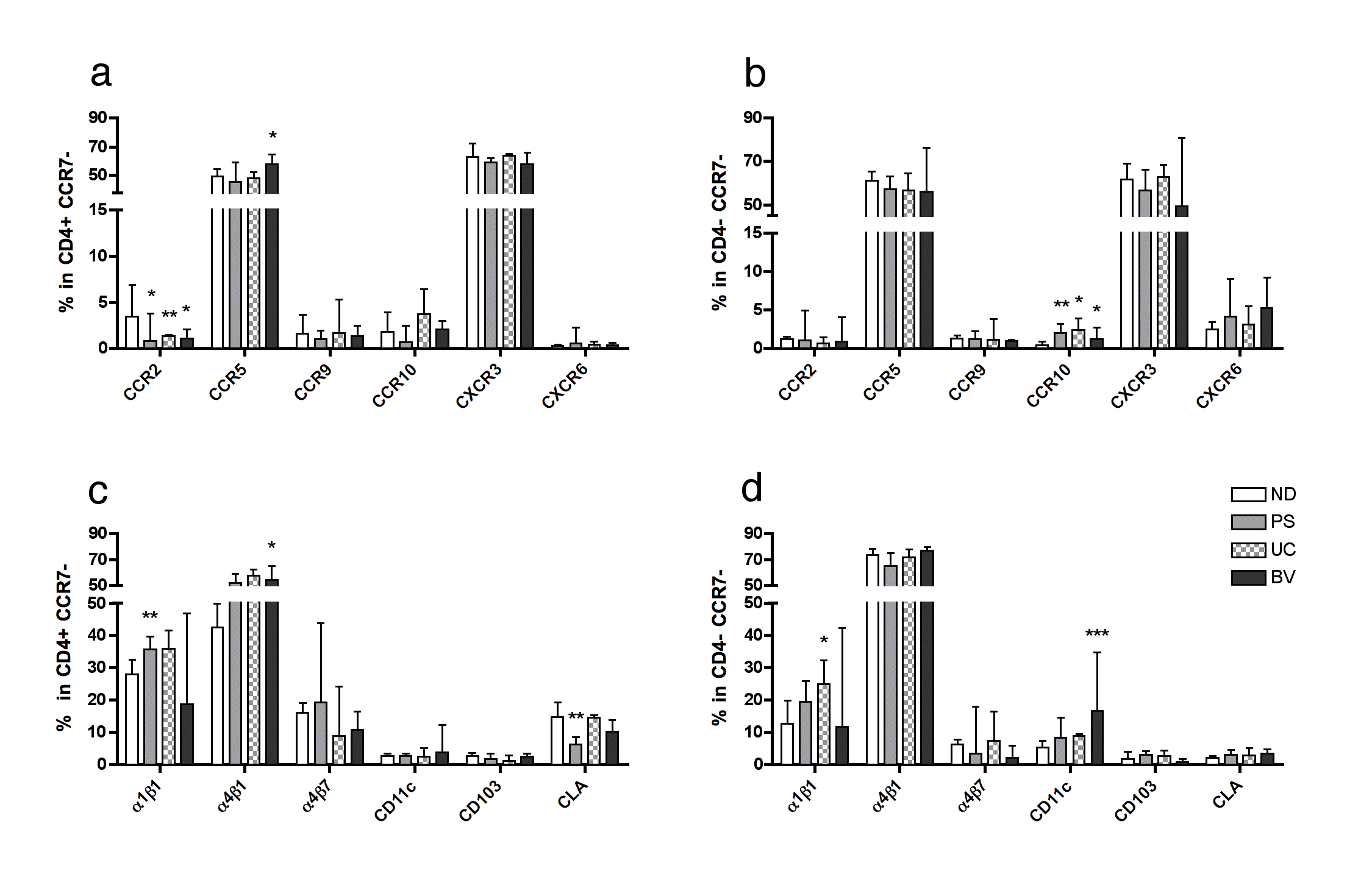

Supplement: S6 Fig — Percentages of the expression of chemokine receptors in total (a) CD4+ effector memory T (TEM) cells and (b) CD8+ TEM cells determined by flow cytometry are shown for normal donors (ND) and the different groups of patients. Percentages of the expression of integrins and other adhesion molecules in total (c) CD4+ TEM and (d) CD8+ TEM cells determined by flow cytometry is shown for ND and the different groups of patients. General gating strategy is shown in Fig 3 and S2 Fig. Each bar represents the median ± interquartile range of healthy young women (ND; white bars, n = 13), women with psoriasis (PS; grey bars, n = 5), ulcerative colitis (UC; checkered bars, n = 4) and bacterial vaginosis (BV; dark bars, n = 5). P values indicate: *<0.05; **<0.01; ***<0.001. (TIF) [file pone.0156605.s006.tif]

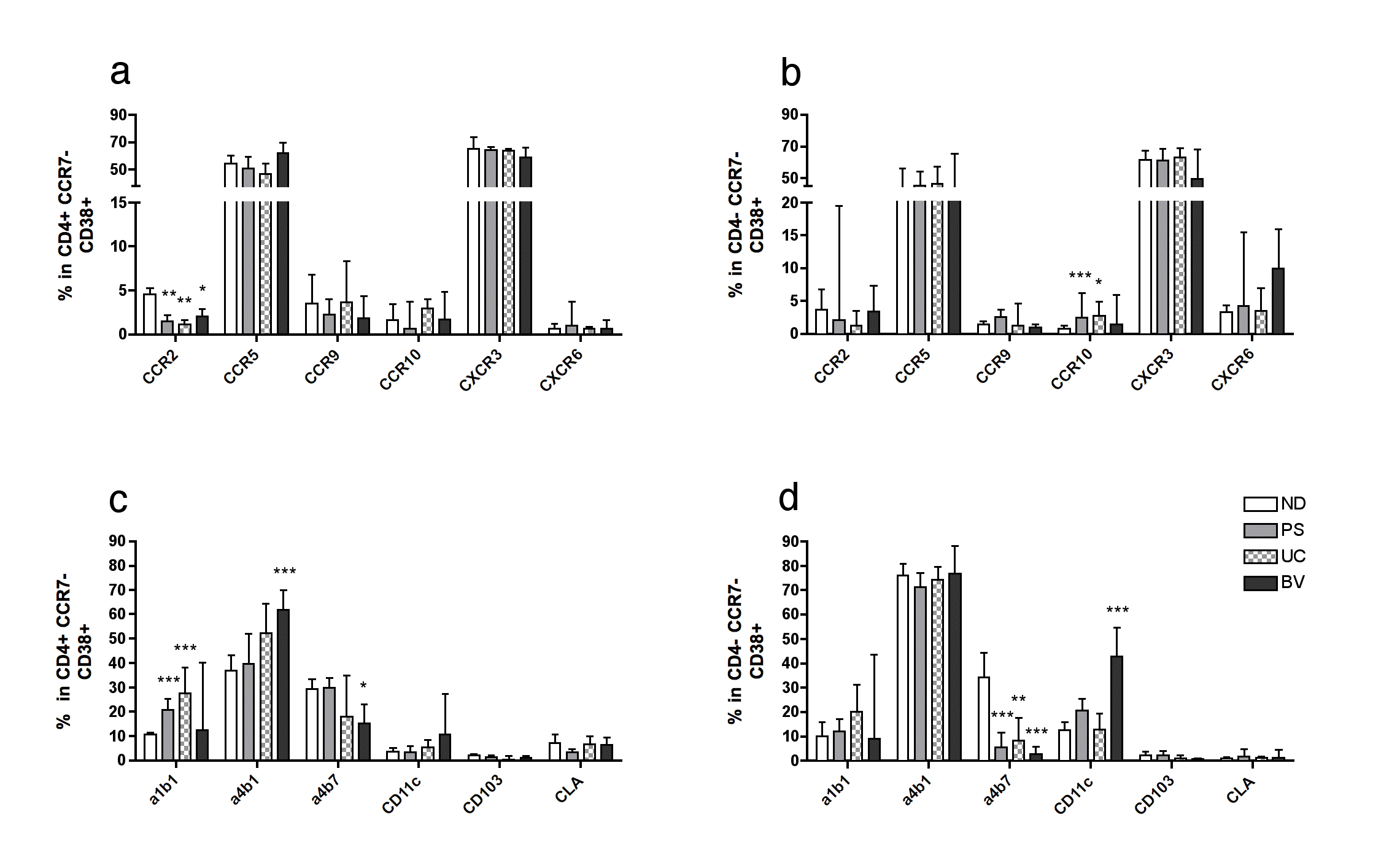

Supplement: S7 Fig — Percentages of the expression of chemokine receptors in CD38+ (a) CD4+ effector memory T (TEM) cells and (b) CD8+ TEM cells determined by flow cytometry are shown for normal donors (ND) and the different groups of patients. Percentages of the expression of integrins and other adhesion molecules in CD38+ (c) CD4+ TEM and (d) CD8+ TEM cells determined by flow cytometry are shown for ND and the different groups of patients. General gating strategy is shown in Fig 3 and S2 Fig. Each bar represents the median ± interquartile range of healthy young women (ND; white bars, n = 13), women with psoriasis (PS; grey bars, n = 5), ulcerative colitis (UC; checkered bars, n = 4) and bacterial vaginosis (BV; dark bars, n = 5). P values indicate: *<0.05; **<0.01; ***<0.001. (TIF) [file pone.0156605.s007.tif]

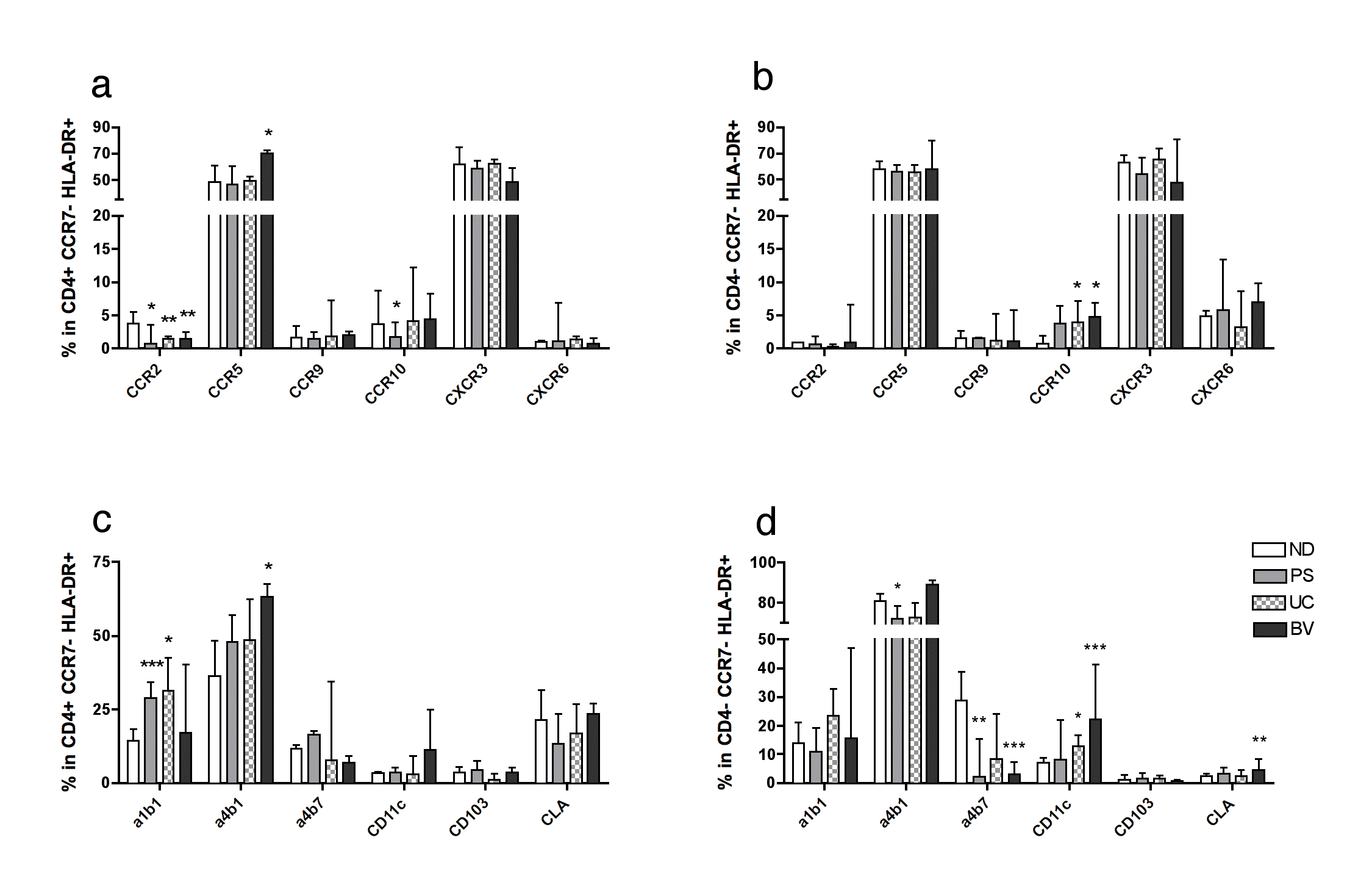

Supplement: S8 Fig — Percentages of the expression of chemokine receptors in HLA-DR+ (a) CD4+ effector memory T (TEM) cells and (b) CD8+ TEM cells determined by flow cytometry are shown for normal donors (ND) and the different groups of patients. Percentages of the expression of integrins and other adhesion molecules in HLA-DR+ (c) CD4+ TEM and (d) CD8+ TEM cells determined by flow cytometry are shown for ND and the different groups of patients. General gating strategy is shown in Fig 3 and S2 Fig. Each bar represents the median ± interquartile range of healthy young women (ND; white bars, n = 13), women with psoriasis (PS; grey bars, n = 5), ulcerative colitis (UC; checkered bars, n = 4) and bacterial vaginosis (BV; dark bars, n = 5). P values indicate: *<0.05; **<0.01; ***<0.001. (TIF) [file pone.0156605.s008.tif]
